# Microfluidic cell sorting by stiffness to examine heterogenic responses of cancer cells to chemotherapy

**DOI:** 10.1038/s41419-018-0266-x

**Published:** 2018-02-14

**Authors:** Muhymin Islam, Roman Mezencev, Brynn McFarland, Hannah Brink, Betsy Campbell, Bushra Tasadduq, Edmund K. Waller, Wilbur Lam, Alexander Alexeev, Todd Sulchek

**Affiliations:** 1 George W. Woodruff School of Mechanical Engineering, Georgia Institute of Technology, 801 Ferst Drive, Atlanta, GA 30332-0405 USA; 2 The School of Biological Sciences, Georgia Institute of Technology, 310 Ferst Drive, Atlanta, GA 30332-0535 USA; 3 Wallace H. Coulter Department of Biomedical Engineering, Georgia Institute of Technology, 313 Ferst Drive, Atlanta, GA 30332-0535 USA; 4 The School of Electrical and Computer Engineering, Georgia Institute of Technology, 313 Ferst Drive, Atlanta, GA 30332-0535 USA; 5 Winship Cancer Institute, Emory School of Medicine, 1365 Clifton NE Rd, Atlanta, GA 30322 USA; 6 Present Address: Office of Research and Development, US EPA, Washington, DC USA

## Abstract

Cancers consist of a heterogeneous populations of cells that may respond differently to treatment through drug-resistant sub-populations. The scarcity of these resistant sub-populations makes it challenging to understand how to counter their resistance. We report a label-free microfluidic approach to separate cancer cells treated with chemotherapy into sub-populations enriched in chemoresistant and chemosensitive cells based on the differences in cellular stiffness. The sorting approach enabled analysis of the molecular distinctions between resistant and sensitive cells. Consequently, the role of multiple mechanisms of drug resistance was identified, including decreased sensitivity to apoptosis, enhanced metabolism, and extrusion of drugs, and, for the first time, the role of estrogen receptor in drug resistance of leukemia cells. To validate these findings, several inhibitors for the identified resistance pathways were tested with chemotherapy to increase cytotoxicity sevenfold. Thus, microfluidic sorting can identify molecular mechanisms of drug resistance to examine heterogeneous responses of cancers to therapies.

## Introduction

Chemotherapy is one of the most common modalities of cancer treatment^1,2^, but its use is complicated by innate and acquired resistance of cancer cells to commonly used anticancer drugs^3^. To address the problem of drug resistance, modern genomic, proteomic, and functional analytical techniques have identified novel genes and signaling networks that determine the responsiveness of tumors to a particular drug treatment^1,2,4,5^. These approaches interrogate clinical samples as a whole and identify molecular signatures and genotypes that predict overall responses to certain drugs. However, determination and prediction of drug response for individual patients is stymied due to complexities caused by cancer cell heterogeneity^1,2,4,5^. Resistance to treatment of a small subset of cancer cells can have a crucial role in cancer progression and disease recurrence in multiple malignancies^6^. The small population of resistant cells can elude chemotherapy in many ways and thus their specific study is needed to identify effectual treatments in precision medicine^7,8^. Since drug-sensitive cells can be orders of magnitude more prevalent than the resistant cells, methods to sort and isolate resistant cells for their study distinct from sensitive cells may enable the discovery of resistance biomarkers and the prediction of alternative treatments to circumvent drug resistance^9,10^. Although fluorescent labels of viability or apoptosis can be used to isolate sensitive and resistant cells, labeling cells with fluorescent tags is time consuming and may alter the properties of the cells and interfere with downstream analyses. For instance, fluorescently labeled caspase inhibitor assay (FLICA)-based reagents not only detect, but also irreversibly inhibit caspase activity, which substantially alters biology of probed cells and seriously limits their use for future studies^11,12^. Therefore, new technologies for label-free functional testing of cells are needed to scrutinize heterogeneous response to drugs.

**Fig. 1:**
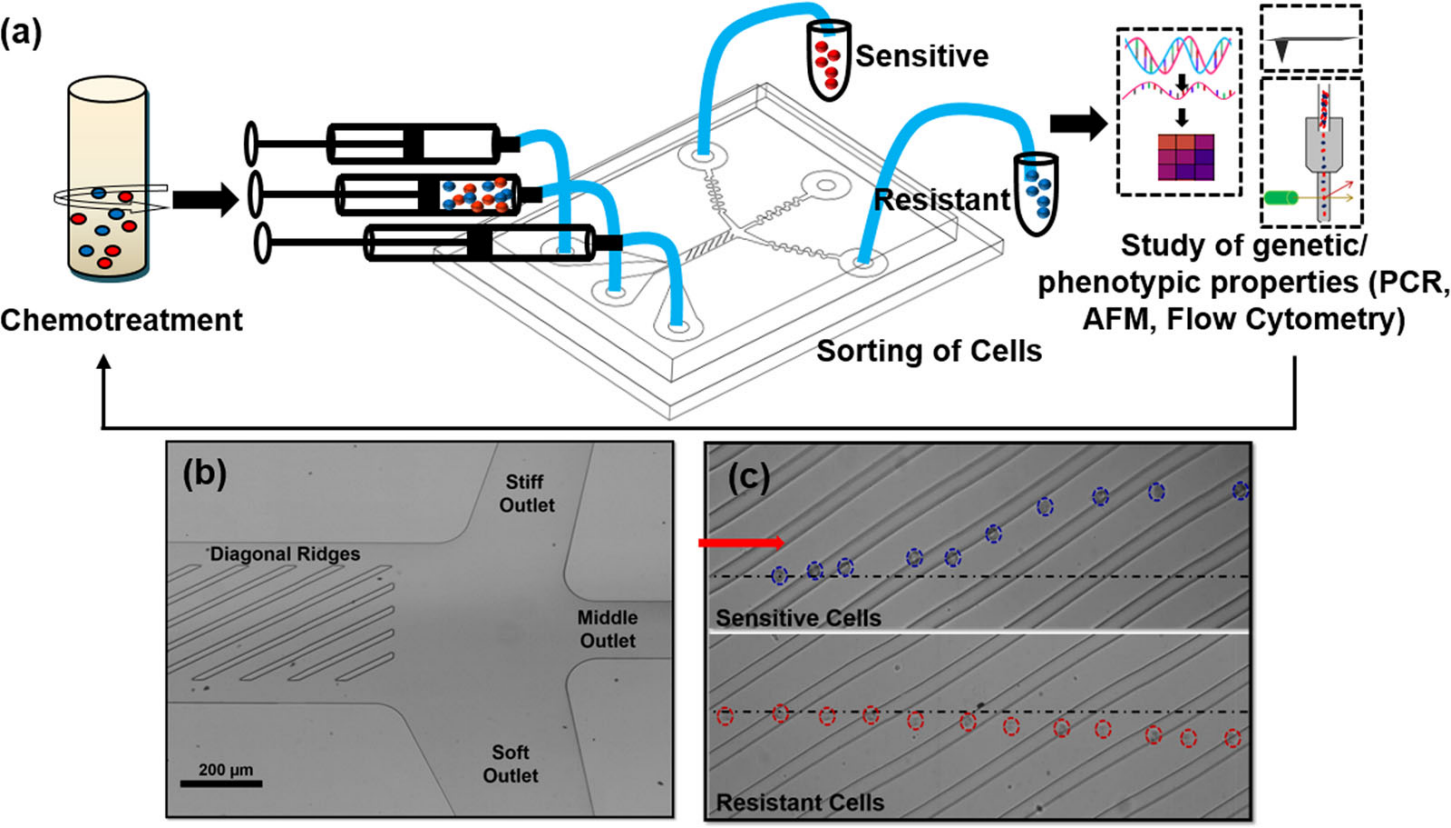
Experimental setup and cell sorting using ridge based microfluidic device. **a** Schematic diagram of the experimental setup showing the sorting of drug-treated cells using microfluidic device and subsequent characterization of gene expression and phenotypic characteristics; **b** optical micrograph of a three-outlet device; **c** representative trajectories of resistant and sensitive cells flowing inside the device

The biophysical properties of cell responses have been effectively exploited previously for sorting and enhanced detection of numerous malignant cells in microfluidic platforms^13–16^, as well as for sorting cells by viability^17^. In this article, a microfluidic device has been used to sort drug-resistant and sensitive leukemia cells by differences in their stiffness that result after treatment with chemotherapy, which was previously identified as an early biophysical response of cells to toxic agents^17–20^. Separated populations were tested to determine their differential gene expression in response to chemotherapy. The microchannel device uses periodic diagonal ridges oriented skew to the direction of fluid flow to compress and sort cells by stiffness and is shown to be highly accurate to separate apoptotic cells^25,26^. The schematic of the process is shown in Fig. 1a and a micrograph of the device is shown in Fig. 1b. Flowing cells are translated perpendicular to the channel axis based on cell biomechanical properties as shown in Fig. 1c.

As a proof of concept, the chemotherapeutic agent daunorubicin was applied to the leukemia cell lines K562 and Jurkat, and a small population of surviving (resistant) cells was isolated using microfluidics. Gene expression differences between sensitive and resistant cells were determined using the quantitative polymerase chain reaction (qPCR). On the basis of a network analysis of gene expression data, several molecular pathways were identified as significant to resistance. Inhibitors of these resistance pathways were then confirmed to increase the cytotoxicity of daunorubicin. Cell stiffness was thus identified as a biomarker that can be used to isolate and study resistant cells. Biophysical sorting introduces a novel opportunity to examine the heterogeneous response of cells to therapies to better address drug resistance and design effective precision treatments against cancer cells.

## Results and discussion

### Characterization of chemotherapy-treated and -untreated cells

**Fig. 2:**
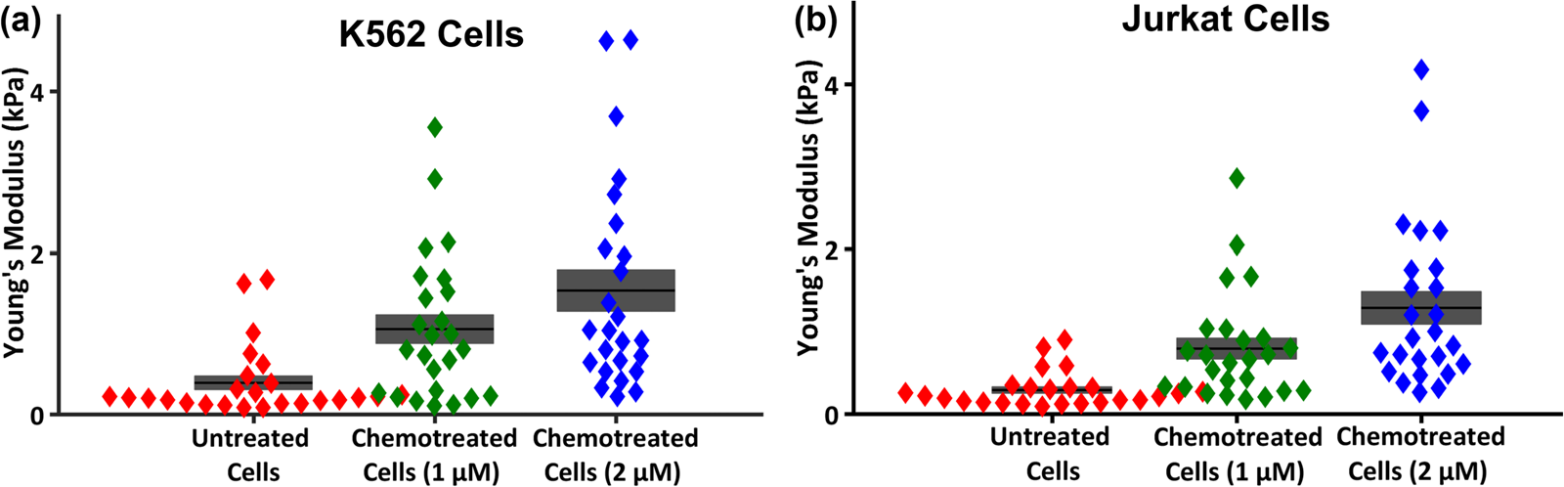
Stiffness of untreated and daunorubicin-treated cells are presented by Young’s moduli. The stiffness of individual cells is shown by individual dots (*N = *25 for each cell type). The bars and shaded regions are representing mean and standard error of mean (SEM), respectively. **a** The stiffness of untreated K562 cells was significantly lower (*p < *0.005) than the stiffness of treated cells for both concentrations of daunorubicin; and **b** the stiffness of untreated Jurkat cells was also significantly lower (*p < *0.001) than stiffness of treated cells for both concentrations of daunorubicin

AFM analysis was conducted on both untreated and daunorubicin-treated K562 and Jurkat cell populations. Cells were treated with 1 µM and 2 µM daunorubicin for ~2 h. The Young’s modulus of K562 and Jurkat cells before and after drug treatment are shown in Fig. 2a and b, respectively. The average Young’s modulus of untreated K562 and Jurkat cells were 0.42 ± 0.38 and 0.29 ± 0.21 kPa, respectively. After 2 µM drug treatment the average Young’s modulus increased threefold to 1.51 ± 1.29 and 1.10 ± 1.08 kPa, respectively (*p*-value < 0.001). The increase of stiffness after the application of chemotherapy is consistent with several previously reported studies^18,21–23^. The stiffness of a cell is associated to the apoptotic response of cells, and include the dynamic changes in the actin cytoskeleton, reduction in cytoplasmic constituents, and cross-linking of the membrane with cytoskeletal structures^18,22,24^.

Untreated and daunorubicin-treated cells were separately flowed through the microfluidics device at various flow rates to optimize their trajectories for sorting experiments. Video microscopy showed that untreated cells followed flow streamlines consistent with cell softness and resulted in a net negative transverse displacement with respect to the direction of fluid flow^25,26^. Untreated (soft) and chemotreated (stiff) cells migrated to opposite sides of the ridged microchannel and sorted according to their differences in mechanical stiffness^14^.

### Sorting of cell mixtures treated and untreated with chemotherapy

**Fig. 3:**
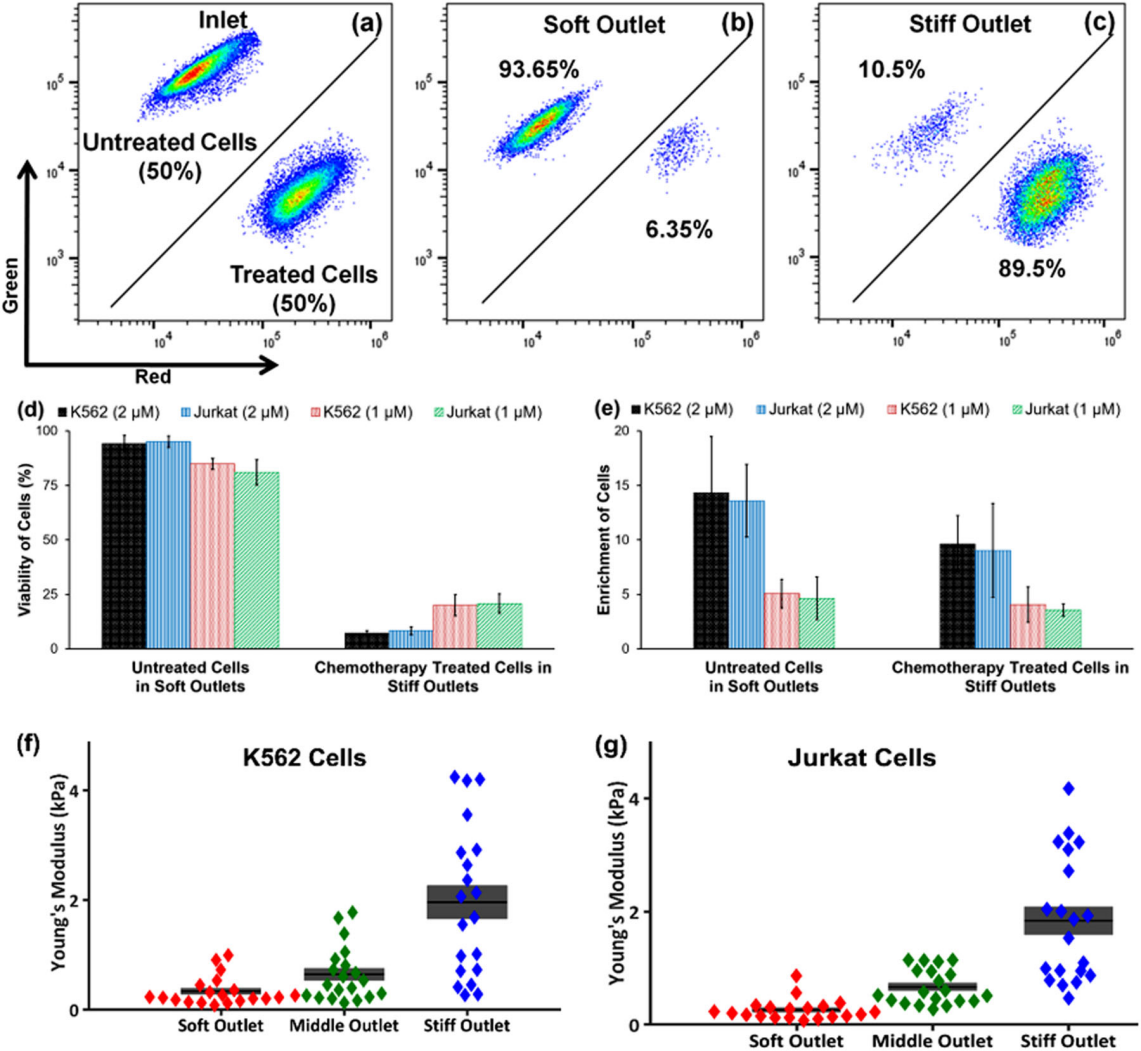
The data obtained by sorting untreated and daunorubicin-treated cells using 3-outlet microfluidic devices. Flow cytometry data from **a** inlet; **b** soft outlet; and **c** stiff outlet; the summarized data of sorted K562 and Jurkat cells at outlets **d** purity of untreated cells in soft outlets and chemotherapy-treated cells in stiff outlets; **e** enrichment of untreated and chemotherapy-treated cells in soft and stiff outlets, respectively, with error bars showing standard deviation (*N = *2). Stiffness of sorted cells from three outlets **f** statistical difference in stiffness of K562 cells among three outlets were significant (*p* < 0.005 between any two outlets, *N = *20); and **g** statistical difference in stiffness of Jurkat cells among three outlets were significant (*p* < 0.001 between any two outlets, *N = *20)

K562 and Jurkat cells were treated with 1 µM and 2 µM daunorubicin for 2 h and stained with red cell tracker. Untreated cells were stained with green cell tracker, and mixed with treated cells of the same type at a ratio of 1:1. The mixture was sorted using the three-outlet microfluidic device at a flow rate of 0.03 ml/min, which was determined to optimize differential trajectories^17^. The device processed ~500 cells/s. The purity of the sorted cells for untreated and daunorubicin-treated cells was dependent on the concentration of daunorubicin for both K562 and Jurkat of cells. For K562 cells and 1 µM drug concentration, the purity was 82.7% of untreated and 80.0% of treated cells in soft and stiff outlet, respectively. For 2 µM concentration, the purity increased to 93.7% and 89.5% in soft and stiff outlet (Fig. 3a–c), corresponding to an enrichment factor of 14.34 and 9.64, respectively, which is consistent with a larger stiffness difference between treated and untreated cells. In the study of Jurkat cells, the purity of recovered untreated and daunorubicin-treated populations of cells (at 2 µM) was found to be 93% and 89% (Fig. 3d, e) with enrichment factor of 13.6 and 9.0, respectively.

### Stiffness of sorted cells

To validate that the sorting mechanism was dependent upon mechanical stiffness, AFM analysis was performed on the sorted cells. After sorting mixtures of untreated and daunorubicin (2 µM)-treated cells, the average Young’s modulus of the cells collected from soft outlets was significantly lower compared to the stiffer outlets (*p*-value < 0.01), as shown in Fig. 3f and g.

### Assessment of apoptosis of sorted cells

**Fig. 4:**
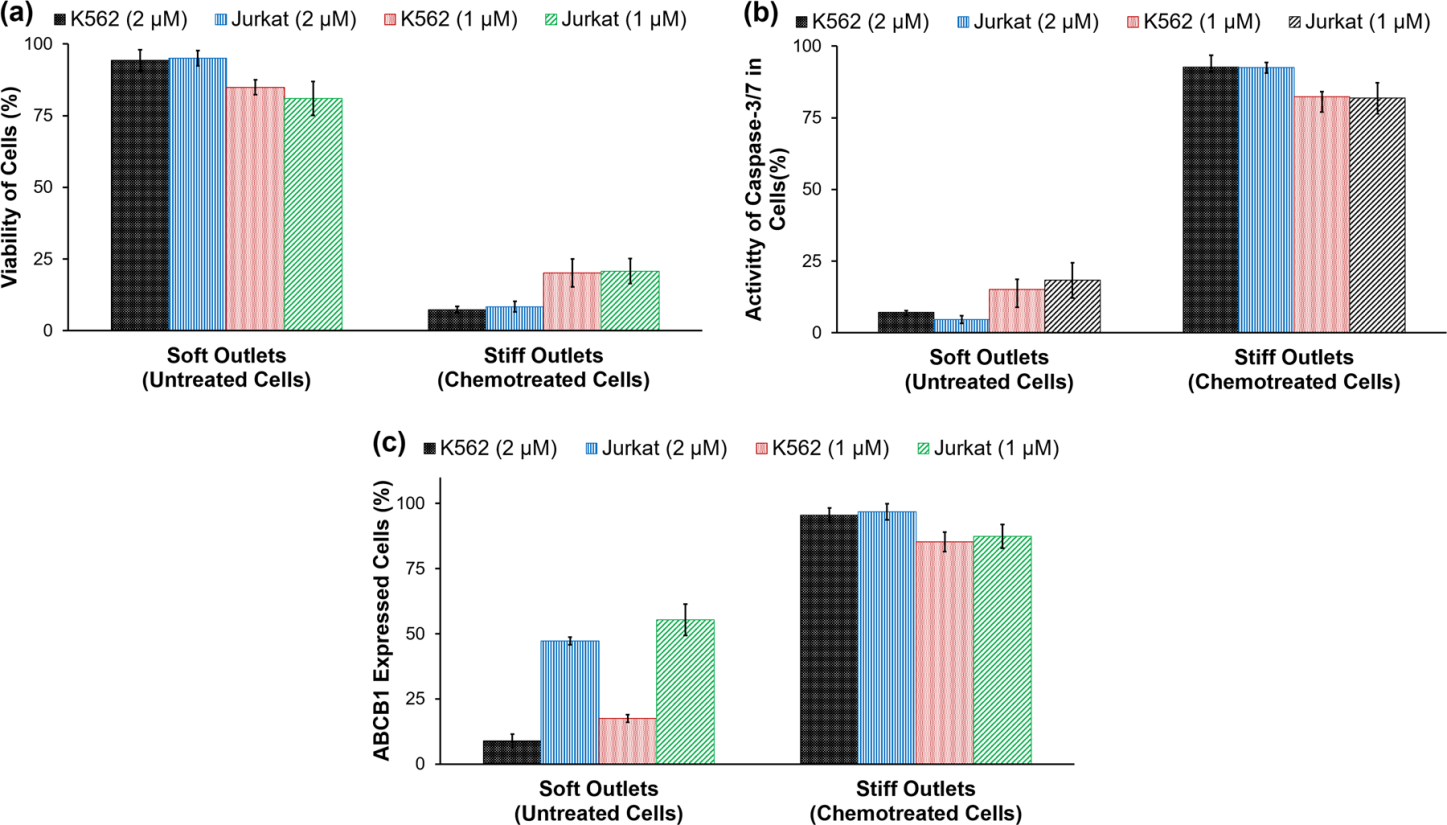
Analysis of sorted cells from soft and stiff outlets for K562 and Jurkat cells using flow cytometry. **a** Viability analysis using EthD-1 stain; **b** activity of Caspase-3/7 gene; **c** Expression of ABCB1 gene. Error bars are showing standard deviation for all the figures (*N* = 2)

K562 cells treated with 2 µM daunorubicin for 2 h were mixed with untreated cells at a ratio of 1:1, and sorted using the 3-outlet microfluidic device. Viability of the cells collected from soft outlet was found to be 94.7% as untreated cells were primarily directed to this outlet. The stiff outlet was found to be enriched for daunorubicin-treated cells and showed a viability of only 7.3%, shown in Fig. 4a. Representative results obtained from flow cytometry analysis are shown in Fig. S1. For the lower concentration of 1 µM, cell viability was 84.9% and 20.15% in soft and stiff outlets, respectively. Similar results of Jurkat cell viability was observed and shown in Fig. 4a. By reducing the concentration of daunorubicin, the decrease in stiffness difference resulted in a decrease in purity of cells in both the outlets and consequently, a lower viability difference in the sorted the cells. The sensitivity and specificity of the device is shown in Table S1 and the diagnostic odd ratio (DOR) is shown in Fig. S2.

Treatment with daunorubicin also induced apoptosis, which was confirmed by increased caspase-3/7 activity (Fig. S3). Daunorubicin causes DNA synthesis inhibition, free radical formation, and lipid peroxidation, DNA binding and the accumulation of DNA damage via the inhibition of topoisomerase II^27^. For both K562 and Jurkat cells, the percentage of cells that showed caspsase-3/7 activity was equivalent to the percentage of nonviable cells, indicating that the nonviable cells followed an apoptotic cell death upon treatment with daunorubicin (Fig. 4b).

### Assessment of ABCB1 expression of sorted cells

ABCB1, a member of the ABC-transporter family responsible for the extrusion of some drugs, is associated with multidrug resistance of cancer cells through aberrant expression of its product MDR1^28,29^. ABCB1 is reported to have low expression in K562 cells which was also observed in this study (Fig. 4c). However, after treating the cells with both the 1 µM and 2 µM daunorubicin, the expression of ABCB1 increased markedly. Untreated and chemotreated cells were mixed at 1:1 ratio and sorted. For K562 cells treated with 2 µM daunorubicin, the ABCB1 expression was detected in 8.95% and 95.45% cells from soft and stiff outlets, respectively (Fig. S4). Similar experiments were performed with Jurkat cells and ABCB1 was expressed in over 40% untreated Jurkat cells. The Jurkat cells treated with 2 µM of daunorubicin and subsequently sorted showed 47.25% and 96.25% cells ABCB1 expression in soft and stiff outlets, respectively. The results are summarized in Fig. 4c.

### Sorting of sensitive and resistant cells after drug treatment

**Fig. 5:**
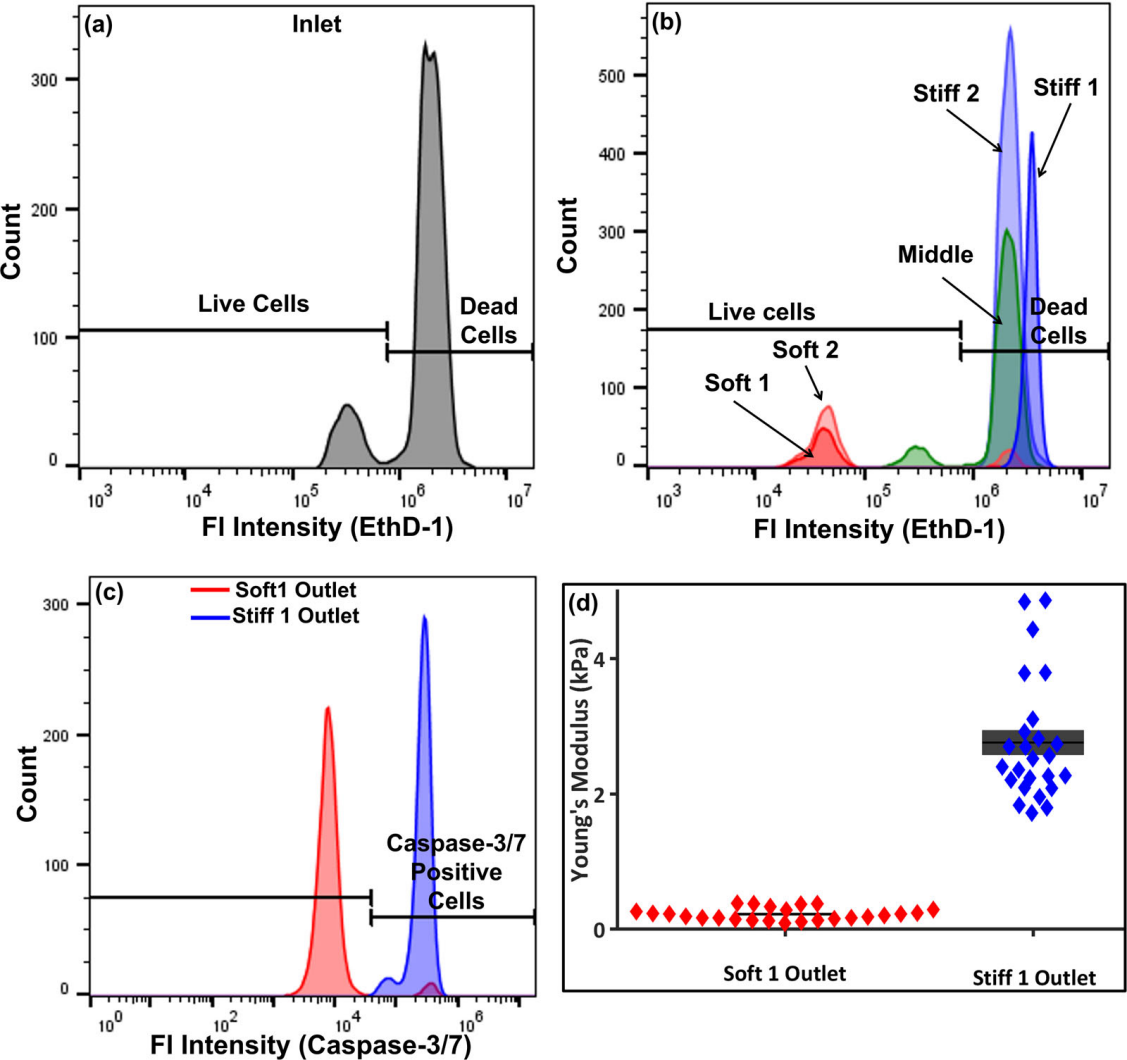
Viability and stiffness analysis of sorted K562 cells after daunorubicin treatment. Viability analysis performed with EthD-1 of sorted K562 cells after treatment with daunorubicin using 5-outlet device, showing the **a** inlet and **b** outlets. **c** Apoptotic marker showing caspase-3/7 activity at different outlets. **d** Stiffness of treated K562 cells after separation to two different outlets (*p* < 0.00001, *N = *25)

K562 cells were treated with a lower dose of daunorubicin (50 nM) for 15 h which resulted in survival of a minority of cells (<15%). The treated cells were then sorted through a 5-outlet device (Fig. 1b) and analyzed using flow cytometry shown in Fig. 5a and b. The 5-outlet design was used to increase the fractionation of the sorted populations to result in both improved sensitivity and specificity^17^. The stiffest outlet (*stiff 1*) had only nonviable cells, whereas the next outlet, *stiff 2*, had 99.7% nonviable cells with an enrichment factor of 59.1. Evaluating the viable cells, the softest outlet *soft 1* enriched viable cells to 96.3% purity with an enrichment factor of 143.52. The next softer outlet, *soft 2*, had 81.8% viable cells with an enrichment of 25.27. The activity of caspase-3/7 was also observed on sorted cells shown in Fig. 5c and the expression of apoptosis markers was consistent with viability results. Daunorubicin-treated K562 cells collected from *soft 1* outlet were also significantly softer than the cells from the *stiff 1* outlet, as measured with AFM (Fig. 5d). This result indicates that minority populations of viable, chemotherapy-resistant cells can be enriched to high purity. A comparison of the improvement in accuracy of the 3-outlet and 5-outlet devices using a DOR analysis^17^ is shown in Fig. S2.

### Gene expression analysis

To observe whether gene expression differences were present between chemotherapy-resistant and sensitive cells, a comparative qPCR analysis was performed upon cells from stiff and soft outlets. The results of gene expressions are summarized in Fig. S5. Apoptosis related genes Casp-3 and Casp-7 were overexpressed in the cells collected from stiff outlet, indicating that the number of nonviable cells were higher in the stiff outlet compared to the soft one^30,31^. Also, the higher expression of KRT-19, a member of keratin family, was observed in the cells collected from soft outlet^31,32^. The keratins are intermediate filament proteins primarily accountable for the structural integrity of epithelial cells. Change in the BCL2 gene was not observed in the cell population after chemotreatment (Fig. S5), but overexpression of this gene in the small subset of soft cells suggested increased resistance to apoptotic cell death^33–35^. From the similarity of the expression profile of cells at inlet and stiff outlets in Fig. S5, we highlight that the sorting allowed us to discover gene expression differences between sensitive and resistant cells separately not observable in the bulk analysis of treated cells.

To understand the underlying mechanism for heterogeneity of responsiveness of cells in the soft and stiff outlets, an array of genes related to cancer drug resistance and metabolism was analyzed. Among 84 examined genes related to drug resistance from the PCR Array, 27 differentially expressed genes were identified, of which 24  were upregulated and 3 downregulated in soft (resistant) cells (Table S3). A sizable group of upregulated genes (9/24) is formed by members of the CYP supergene family that encode cytochrome P450 monooxygenases (CYP450). CYP450 enzymes are responsible for 80% of phase I drug oxidation reactions^36^, and these reactions can activate or inactivate numerous anticancer drugs. For instance, CYP3A4, which metabolizes about half of all marketed drugs, was found upregulated in soft/resistant cells. CYP3A4 and CYP2C8, which are both upregulated in soft cells, are major CYP450 enzymes responsible for oxidation and inactivation of anticancer drug taxol^37^.

The finding of upregulated CYP1A1 and CYP1A2 in soft (resistant) cells is consistent with the findings^38^ that CYP450 genes are significantly upregulated in doxorubicin-resistant cells developed from a MCF-7 breast cancer cell line. Upregulation of antiapoptotic gene BCL2 and downregulation of pro-apoptotic gene BAX, detected in soft (resistant) vs. stiff (sensitive) cells, are known to desensitize various cancer cells to apoptosis, which is consistent with a drug-resistant phenotype^39^. Another gene identified as upregulated in soft (resistant) cells is CDKN1A, which encodes a cyclin-dependent kinase inhibitor p21^(WAf1/CIP1)^. Upregulation of CDKN1A was previously shown to protect colon cancer cells against apoptosis induced by doxorubicin (a structural analog of daunorubicin) through inhibition of caspase-3 activation^40^. Interestingly, upregulation of CDKN1A also reportedly inhibits apoptosis induced in chronic myelogenous leukemia (CML) cells upon treatment with targeted therapeutic agent imatinib^41^.

Upregulation of transporter gene ABCC1 was detected in soft (resistant) cells relative to stiff cells. This gene encodes multidrug resistance-associated protein 1 (MRP1), an ABC transporter with unusually broad substrate specificity^42^. MRP1 is capable of extruding a wide variety of neutral hydrophobic compounds and contributes in this way to the defense against xenobiotics, endogenous toxic metabolites, and oxidative stress.

EGFR was found upregulated in soft (resistant) cells. Deregulation of EGFR through various mechanisms, including overexpression of EGFR gene, has been demonstrated in various solid tumors and associated with poor prognosis in some tumor types^43^. EGF also acts as a survival factor, and deregulated EGFR-signaling inhibits apoptosis through downstream effectors PI3K/Akt and MEK/Erk^44^. Inhibition of EGFR would be reasonably expected to reverse the observed deregulation of Bcl-2 and Bax and increase drug sensitivity of resistant cells^45^.

The inverse association between CYP3A4 and ABCB1/MDR1 is intriguing. Specifically, resistance to daunorubicin in leukemia cells is associated with upregulation of ABCB1 gene^46^, while the soft (resistant) cells in our study display downregulation of the ABCB1 gene, which encodes for the multidrug resistance protein 1 (MDR1, P-glycoprotein). However, downregulation of MDR1 in soft cells does not necessarily imply increased sensitivity to daunorubicin because increased expression of CYP3A4 is likely enhancing metabolism and inactivation of the drug in soft cells. Most compounds that are substrates of CYP3A4 are also substrates or inhibitors of MDR1^47^. In addition, both CYP3A4 and ABCB1 gene encoding for MDR1 protein are transcriptionally regulated by pregnane X receptor (PXR) that acts as a xenobiotic sensor regulating various phase I and phase II drug metabolizing enzymes and transporters^48^. Thus, the downregulated *ABCB1* gene does not imply increased sensitivity of cells to MDR1 substrates as these agents can be metabolized and inactivated by upregulated CYP3A4. Consequently, upregulation of CYP3A4 may counteract downregulation of MDR1, as both these resistance factors are known to protect cells against many of the same cytotoxic agents.

To identify high-level regulators of gene expression that can be responsible for the observed differences in gene expression between soft (resistant) and stiff (sensitive) cells, we performed various systems level analyses that included interactome analysis, network building and topological scoring with pathway enrichment analysis. Interactome analysis identified 127 transcription factors in the entire MetaCore human protein interaction network (*N* = 40,221 network objects) that display significant overconnectivity to 27 genes differentially expressed between soft and stiff cells (Supplemental File 1). At least some of these 127 overconnected transcription factors, selected with stringent criteria (FDR = 0.001), represent regulator genes likely involved in gene expression changes observed between soft and stiff cells. Of them, three transcription factors: ESR1, NF-kB, and PPARA are overconnected, as well as upregulated, in the soft vs. stiff cells.

**Fig. 6:**
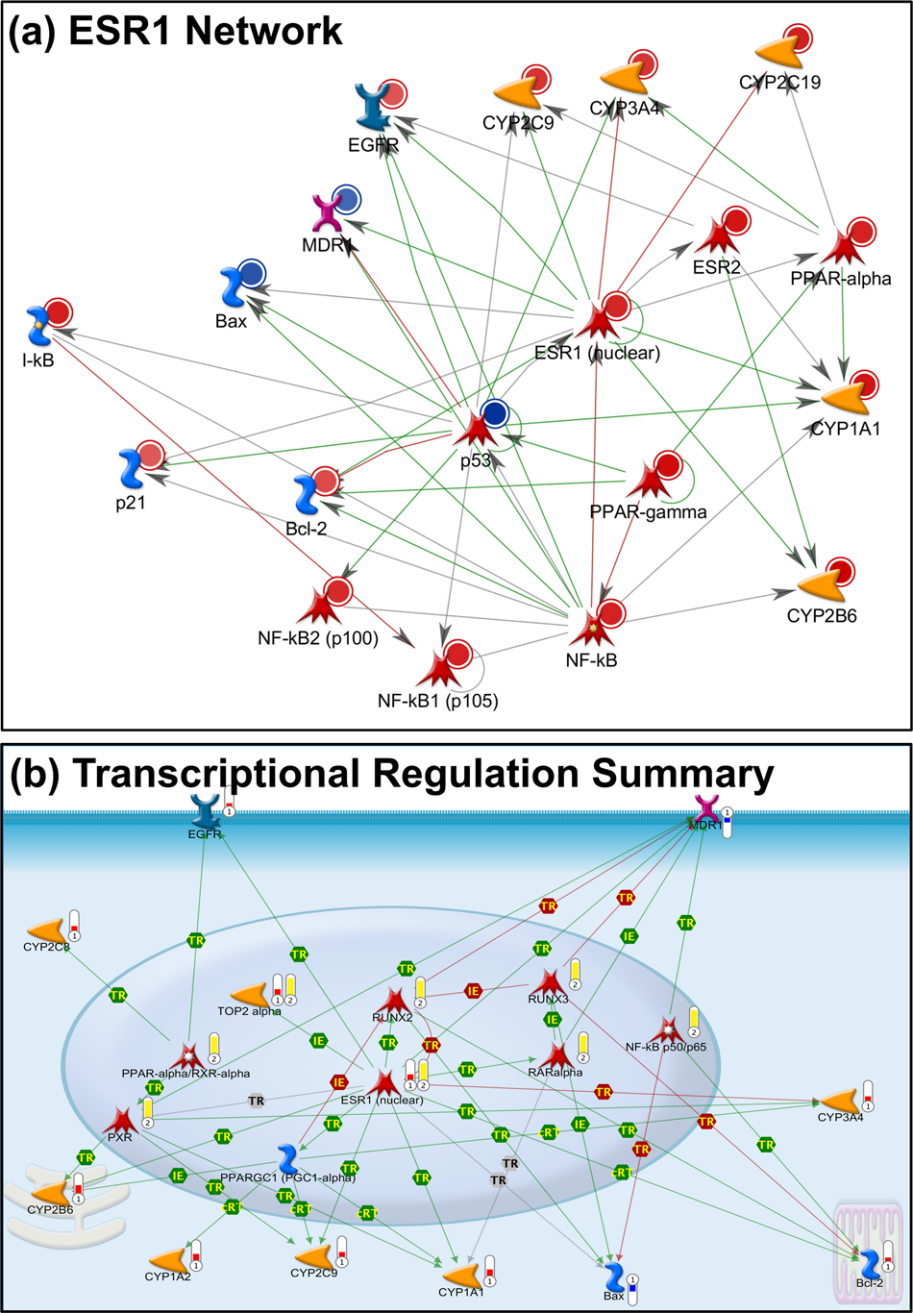
Gene expression analysis of resistant and sensitive cells. **a** Transcription factor-centric network around ESR1 built from genes differentially expressed between soft and stiff cells. Red circle: upregulated gene; blue circle: downregulated gene; green edge: transcriptional activation; red edge: transcriptional repression; **b** custom map produced from genes differentially expressed between soft and stiff cells and topologically relevant genes for differentially expressed set. Edges represent direct transcriptional regulation or influence on expression between map objects. Red thermometer: genes upregulated in soft (resistant) cells; blue thermometer: genes downregulated in soft (resistant) cells; yellow thermometer: genes topologically relevant to set of upregulated genes in soft (resistant) cells (for additional legend: https://portal.genego.com/legends/MetaCoreQuickReferenceGuide.pdf)

The implied relevance of ESR1 (estrogen receptor 1; ERα) in soft cells is unexpected considering the fact that this receptor is usually involved in sex-specific tissue cancers (breast, ovarian, endometrial, and prostate). Building networks from 27 differentially expressed genes using the “Transcription Regulation” algorithm produced a prioritized list of 30 transcription factor-centric networks (Supplemental File 2). Intriguingly, the network centered on ESR1 (Fig. 6a) scores among the top of these prioritized transcription factor-centric networks. This finding further implicates estrogen receptor 1 in the observed gene expression changes. Regarding ESR1, a prior gene expression study of chronic myelogenous leukemia (CML) has uncovered that genes upregulated in CML were significantly enriched with genes regulated by estrogen receptor. The effect of estrogen signaling was seen in a data set that included CML specimens from men and women of various ages. In addition, the enrichment for ERα-regulated genes was seen in a subset of known CML-associated genes. Taken together, these findings implied the role of estrogen receptor activity in CML, even in men and postmenopausal women^49^.

Upregulation of genes NFKB1 and NFKB2, encoding NF-κB family members p100/52 and p105/50, was found in soft cells. Activation of NF-κB signaling is known to occur upon treatment with anticancer drugs and cause resistance in cancer cells^50^, but upregulation of NFKBIB^51^ and NFKBIE^52^ genes in soft cells, which both encode inhibitors of NF-κB signaling, lowers the confidence in the role of NF-κB signaling in resistance of soft cells. Nevertheless, activation of NF-κB signaling is supported by results of interactome analysis and transcription regulation network building (Supplemental Files 1 and 2).

Interestingly, soft cells in our study also displayed upregulation of ESR2 gene that encodes ERβ receptor. Significant enrichment of pathway map “Regulation of actin cytoskeleton by Rho GTPases**”** further supports the role of differences in actin cytoskeleton between soft and stiff cells in our study (Fig. S6).

Taken together, the data suggest that activation of ESR1 (ERα) can be directly or indirectly involved in several differences in gene expression observed between soft and stiff cells (Fig. 6b). The expression signature of ER-signaling has been found in CML specimens^49^, and activation of ER-signaling reportedly decreased stiffness of osteoblasts and endothelial cells^53,54^. Other differences in gene expression can be attributed to genes identified as topologically relevant or overconnected to the set of our differentially expressed genes (Fig. 6b; Supplemental File 1). Our results suggest a possible role of multiple mechanisms involved in the drug resistance of soft cells, including decreased sensitivity to apoptosis, enhanced phase I metabolism of anticancer drugs and their enhanced extrusion from cells by an ABC transporter. Nevertheless, ERα seems to be critically involved in at least some of these diverse mechanisms and its modulation may at least in part decrease drug resistance and increase cell stiffness, which can result in cancer cells that display less resistant/less aggressive phenotype. Additional details of the gene expression data are described in the Supplement “Gene Expression Pathways” section.

### Experimental validation of the role of ESR1, NF-κB, and CYP3A4 in the resistance of K562 cells

**Fig. 7:**
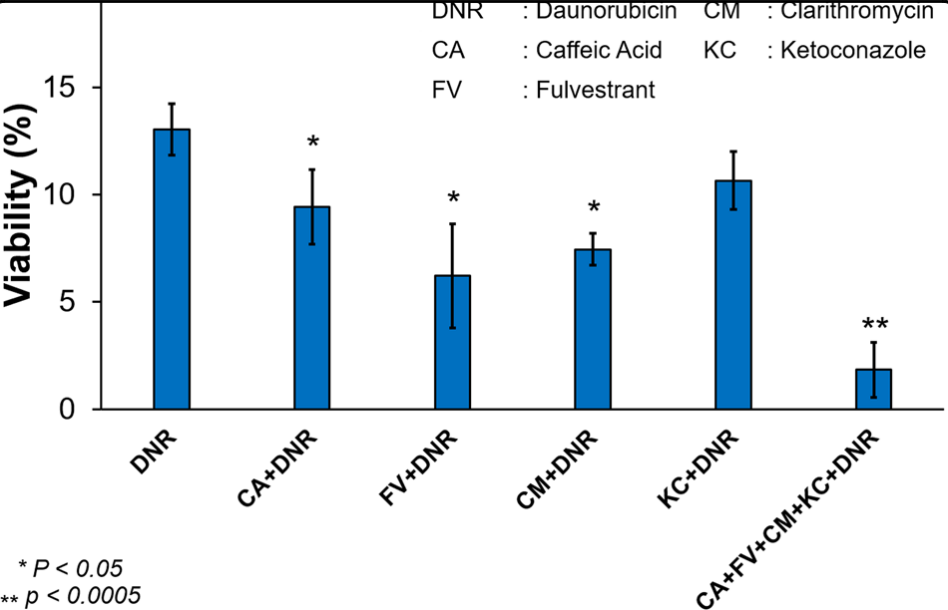
Viability of inhibitors and chemotherapy treated K562 cells. Average viability with standard deviation of inhibitors and chemotherapy-treated K562 cells after (*N = *3), with *p*-values are calculated with respect to only daunorubicin-treated cells in which * represents *p* < 0.05 and ** represents *p* < 0.0005

To experimentally validate the pathways identified by gene expression and in silico analyses as potentially responsible for drug resistance of K562 cells, including ESR1, NF-κB, CYP3A4, and PXR, products of genes were inhibited by treatment of cells with low-molecular weight inhibitors at concentrations/times that did not influence cell viability. Cells with inhibited ESR1, NF-κB, or CYP3A4  activity subsequently displayed significantly increased sensitivity to cytotoxic effects of daunorubicin (Fig. 7), reducing the number of surviving cells up to sevenfold, and supporting our prediction of the role of identified pathways in drug resistance of leukemia K562 cells. The control experiments of the nontoxic antagonist treatments are shown in Fig. S7. Thus, the microfluidic sorting of resistant cells can potentially solve the unmet challenge in individualized therapy to choose supporting agents in combination therapy with improved activity against dysregulated pathways in leukemic cells to produce long-lasting remissions^55^.

Here we showed that differential cell stiffness can be an effective biomarker for rapid and non-destructive separation of resistant cells from sensitive leukemia cells after chemotherapy treatment for comparative analysis of their genetic/phenotypic properties and determination of the underlying mechanisms through network analyses. The purity of the isolated resistant cells was over 95%. Thus, microfluidics processing can examine gene expression differences between sensitive and resistant cells accurately in spite of the small initial percentages of resistant cells. The plausible mechanisms related to drug resistance were identified. The roles of estrogen receptor signaling, NF-κB signaling and CYP3A4 activity in resistance of K562 cells have been experimentally validated by testing  their inhibitors in combination with chemotherapy to reduce drug resistance. As a result, cell sorting by biophysical properties can be used to examine heterogeneous responses of cells to chemotherapy treatments with possible future application in precision medicine approaches to improve chemotherapy selection and use.

## Materials and methods

### Fabrication of microfluidic device

The microfluidic device was fabricated using replica molding of polydimethylsiloxane (PDMS) on a SU-8 patterned silicon wafer^13–15^. All devices tested were designed in AutoCAD and simulated the flow trajectories in ANSYS Fluent. The molds for the device were fabricated on a silicon wafer by spin coating SU-8 2007 (SU-8 2007, Microchem Corp.) using a two-layer photolithography process. The dimensions of the molds, particularly the ridge heights, were measured with profilometry (Dektak 150 profiler) and optical microscopy. Several device parameters influence cell trajectories, which include ridge gap distance, number of ridges, inter-ridge spacing, and angle of ridges. The effects of ridge angles, ridge gap, ridge spacing, and number of ridges was studied previously^13–15^. The ridge angle, ridge number, ridge spacing, and ridge gap were chosen to be 30°, 14 ridges, 200 µm, and 9 µm, respectively^17^. The ridge gap was chosen to be small enough to compress the cells sufficiently without clogging the device and compares to an average cell diameter of 15 µm. Three and five outlet devices were tested to evaluate the accuracy of fractionation of the heterogeneous cells to isolate target cell types. The mold pattern was translated to polydimethylsiloxane (PDMS), inlet and outlet holes were punched with biopsy punch, and the chip bonded to glass^13,14,17^. To prevent non-specific cell adhesion to the microfluidic channel walls, the device was coated with bovine serum albumin (Sigma Aldrich) at a concentration of 10 mg/ml and incubated at 4 °C overnight.

### Cell culture and treatments

Jurkat (CRL-1990) and K562 (CCL-243) cells were purchased from ATCC. The cells were cultured and maintained in RPMI-1640 medium (Sigma) with the addition of 10% FBS and 1% penicillin streptomycin. All cells were incubated at 37 °C in humidified air with 5% CO_2_. Cells were expanded to 80% confluency in non-treated cell culture flasks over two days. Cells were treated with daunorubicin at concentrations of 0.05 µM, 1 µM, and 2 µM for 2 and 15 h of exposure.

### Experimental setup

The accuracy of sorting was tested by mixing fluorescently labeled daunorubicin-treated cells with untreated cells, sorting using the microfluidic device, and analyzing the outlets using flow cytometric analysis. Gene expression differences of resistant cells were determined by treating all cells with daunorubicin and sorting using microfluidics. In all cases, cells were washed and resuspended in PBS at a concentration of ~1 million cells/ml and infused into the microfluidic device using a syringe pump (PHD 2000, Harvard Apparatus) at specified flow rates. The device flow was formed by three inlet streams, which included two sheath streams to hydrodynamically focus the stream containing the cells. The cell trajectories were observed by an inverted bright-field microscope (Eclipse Ti, Nikon) and recorded by high-speed camera (Phantom v7.3, Vision Research) at a frame rate of 2000 frames per second^13,17^. The stiffness of cells before and after separation experiments was measured using atomic force microscopy (AFM, MFP-3D, Asylum Research^56,57^) in suspension-like condition to retain rounded morphology and similar indentation range as microfluidics. To improve cell stability during the AFM measurement, a monolayer of Cell-Tak (BD Biosciences) was applied to gently attach cells to the glass substrate. Beaded silicon nitride cantilevers (spring constant 37.1 pN/nm) were used to indent the center of the cells at a rate of 1.5 μm/s. Sufficient force was applied to achieve at least 5 μm deformation such that compression was in close comparison with the microfluidic experiments. Cell Young’s modulus values were calculated from the force-indentation curves and fit to a Hertzian model to compute the average Young’s modulus. One-way analysis of variance (ANOVA) was performed between Young’s modulus of chemotherapy-treated and -untreated cells to determine statistical significance.

### Flow cytometry analysis

To differentiate in flow cytometry, cells were labeled with 2 µM with CellTracker™ red (chemotherapy-treated cells) and green (untreated cells) (Molecular Probes Inc.) for ~1 h in 37 °C. After loading the cells with the dye, the accuracy of sorting could be quantified. From flow cytometry results, the enrichment factor was calculated from that using the following equation:

$$\frac{{\left( {{\mathrm{Number}}\,{\mathrm{of}}\,{\mathrm{green}}\,{\mathrm{cells}}/{\mathrm{Number}}\,{\mathrm{of}}\,{\mathrm{red}}\,{\mathrm{cells}}} \right)_{{\mathrm{Outlet}}}}}{{\left( {{\mathrm{Number}}\,{\mathrm{of}}\,{\mathrm{green}}\,{\mathrm{cells}}/{\mathrm{Number}}\,{\mathrm{of}}\,{\mathrm{red}}\,{\mathrm{cells}}} \right)_{{\mathrm{Inlet}}}}}.$$

The sensitivity and specificity at different outlets, as well as the DOR was determined as described previously^17,57^. The sensitivity measures the proportion of positive cells that are correctly identified and specificity measures the proportion of negatives that are correctly identified from a sample. A confusion matrix was used to determine condition positive outcomes, which were untreated cells in the in the soft outlet and treated cells in stiff outlet, whereas negative outcomes were defined as untreated cells in stiff outlet and treated cells in soft outlet, resulting in frequencies of true positives, false positives, false negatives, and true negatives (TP, FP, FN, and TN, respectively). Cell viability of microfluidic sorted cells and control cells that were not processed by microfluidics was tested by flow cytometry analysis using 2 µM ethidium homodimer-1 (EthD-1) (Molecular Probes Inc.)^17,58,59^. EthD-1 is a cell impermeable nucleic acid stain that shows strong fluorescence at 635 nm when bound to DNA in dead cells with disintegrated cell membranes. Apoptosis was determined by flow cytometry analysis through activation of caspase-3/7 using Cell Event Caspase-3/7 Green flow cytometry assay kit (Invitrogen)^60,61^. To determine the expression of the ABC-transporter protein ABCB1, cells were incubated with P-glycoprotein antibody (UIC2) conjugated with PE (ThermoFisher Scientific) for 1 h, washed, and resuspended in PBS to analyze by flow cytometry^28,62,63^. Flow cytometry analysis was performed using BD Accuri C6 flow cytometer (BD Biosciences).

### qPCR

For chemosensitive and chemoresistant cells, ~10,000 cells were collected from stiff and soft outlets and total cellular RNA was isolated using a RNA isolation kit (Macherey-Nagel) according to the instructions provided by the manufacturer. Thereafter, RNA was reverse-transcribed to cDNA using the kit purchased from ThermoFisher Scientific (Catalog number 4387406) following the manufacturer’s instruction. qPCR was used to analyze expression of transporter, apoptosis, structural integrity, and resistance related genes using the 2×TaqMan® PreAmp Master Mix (Applied Biosystems, PN 4391128) and Fluidigm Biomark system. The primers were designed using Primer 3 Plus website (http://www.bioinformatics.nl/cgi-bin/primer3plus/primer3plus.cgi) and “BLAT” tool from UCSC genome browser website (http://genome.ucsc.edu/). The list of primers is given in Table S2.

In addition, expression of genes related to apoptosis, xenobiotic metabolism and drug resistance was investigated using a commercially available PCR array ExProfile™ Human Cancer Drug Resistance & Metabolism Related Gene qPCR Array (Catalog Number: QG007-B6, Genecopedia, Inc.), using the StepOnePlus^TM^ instrument from Applied Biosystems. The array tested for expression of 84 genes relevant to drug resistance and drug metabolism, as well as endogenous control genes. GAPDH gene was used for normalization of gene expression. Soft and stiff sub-populations separated from the same daunorubicin-treated populations of K562 cells were analyzed as matched-pairs. Genes that were selected as differentially expressed showed a two-tailed *p*-value < 0.05 using the matched-pair *t*-test and an absolute fold change (FC) values ≥ 1.5. The use of multiplicity correction by FDR found that *q*-values ≤ 0.074 for all genes selected as significantly differentially expressed.

### Identification of resistance pathways with systems biology analysis of gene expression

Differentially expressed genes were analyzed with MetacoreTM suite v 6.31 build 68930 (Thomson Reuters) using the following approaches: (i) enrichment analysis in GeneGO canonical “Pathway Maps” functional ontology, (ii) build networks using “Transcription Regulation” algorithm, and (iii) interactome overconnectivity (one-step) analysis for transcription factors. One-way analysis of variance (ANOVA) was performed between soft and stiff outlets to determine statistical significance in gene expressions. The topological significance analysis (TSA) of gene expression profile was performed using online tool provided by GeneGO, Inc. (http://topology.genego.com/zcgi/topology_scoring.cgi). Enrichment analysis was employed to identify GeneGO signaling pathway maps significantly enriched by differentially expressed genes. Transcriptional regulation network building tool produces networks with central transcription factors connected to several differentially expressed genes largely one-step away. One-step interactome analysis for transcription factors determines the relative connectivity of all known transcription factors from MetaCore global interactome to the set of differentially expressed genes and identifies direct neighboring transcription factors with significant connectivity^64^. TSA further extends one-step interactome analysis and identifies “hidden nodes” as genes that occupy topologically significant positions in MetaCore global interactome with respect to differentially expressed genes (without limiting to local interaction neighborhood)^65,66^. Topologically significant genes (*p* < 0.01) were identified for all genes upregulated in soft cells relative to stiff cells using the “transcriptional activation paths from all nodes” algorithm and subsequently mapped to GeneGO human signaling pathway maps as described above. Multiple testing correction was performed using false discovery rate with the adaptive threshold set to permit no more than one pathway incorrectly predicted as significantly enriched. Insight generated from analytical approaches described above was used to build a custom signaling map representing simplified model that could explain changes in gene expression observed between soft and stiff cells (Map Editor tool in MetaCore; Thomson Reuters).

### Effect of ESR1, NF-κB, and CYP3A4 on the resistance of K562 cells

The effect of inhibition of genes identified as likely involved in differences between soft and stiff cells on anticancer activity of daunorubicin was tested using specific small molecule inhibitors. K562 cells were treated with caffeic acid phenethylester (inhibitor of NF-κB, 5 µM), fulvestrant (selective estrogen receptor downregulator, 1 µM), clarithromycin (CYP3A4 inhibitor, 25 µM), and ketoconazole (PXR inhibitor, 1 µM) (Abcam) for 15 h and then the viability of cells was assessed. Concentrations of these inhibitors were selected based on preliminary results showing no significant loss of viability in K562 cells treated with the inhibitors alone at these concentrations. For combined treatment with all four inhibitors together, the concentration was optimized for caffeic acid phenethylester, fulvestrant, clarithromycin, and ketoconazole as 1.2, 0.25, 6.2 and 0.25 µM, respectively. Cells have been experimentally validated by testing antagonists in combination with chemotherapy to reduce drug resistance. As a result, cell sorting by biophysical properties can be used to examine heterogeneous responses of cells to chemotherapy treatments with possible future application in precision medicine approaches to improve chemotherapy selection and use.

### Disclaimer

The views expressed in this paper are those of the author(s) and do not represent official EPA policy.

## Electronic supplementary material

Supplemental Materials
