# Supplementary material for: Microfluidic cell sorting by stiffness to examine heterogenic responses of cancer cells to chemotherapy
Source: Cell Death Dis. 2018 Feb 14;9(2):239. doi: 10.1038/s41419-018-0266-x (PMC5833447; doi:10.1038/s41419-018-0266-x)
Supplement: Supplementary file 1 — Supplemental Materials [file 41419_2018_266_MOESM1_ESM.docx]

**Mcrofluidic Cell Sorting by Stiffness to Examine Heterogenic Responses of Cancer Cells to Chemotherapy**

Muhymin Islam^a^, Roman Mezencev^b^^[[1]](#footnote-1)^, Brynn McFarland^c^, Hannah Brink^c^, Betsy Campbell^c^, Bushra Tasadduq^d^, Edmund K. Waller^e^, Wilbur Lam^c^, Alexander Alexeev^a^, and Todd Sulchek^*a,c^

^a^George W. Woodruff School of Mechanical Engineering, Georgia Institute of Technology, 801 Ferst Drive, Atlanta, GA, 30332-0405, USA

^b^The School of Biological Sciences, Georgia Institute of Technology, 310 Ferst Drive, Atlanta, GA, 30332-0535, USA

^c^Wallace H. Coulter Department of Biomedical Engineering, Georgia Institute of Technology, 313 Ferst Drive, Atlanta, GA, 30332-0535, USA

^d^The School of Electrical and Computer Engineering, Georgia Institute of Technology, 313 Ferst Drive, Atlanta, GA, 30332-0535, USA

^e^Winship Cancer Institute, Emory School of Medicine, 1365 Clifton NE Rd: Atlanta, GA 30322

*E-mail: [todd.sulchek@me.gatech.edu](mailto:todd.sulchek@me.gatech.edu)

**Supplementary Information**

**
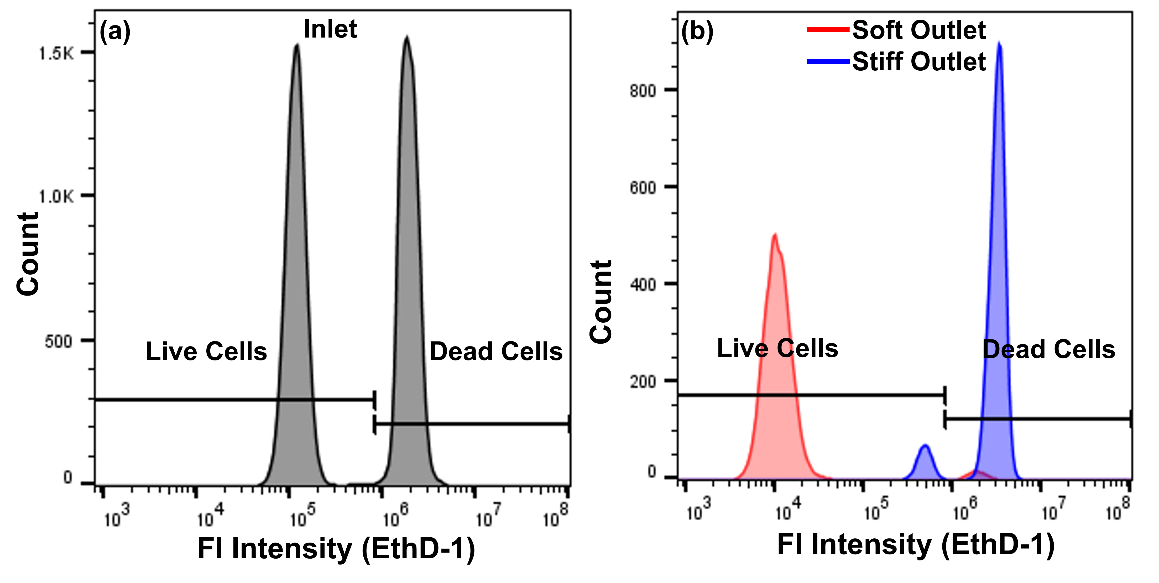
**

Fig. S1: Viability study of the sorted cells using EthD-1 (K562 cells treated with 2 µM daunorubicin). Flow cytometry data from (a) inlet; (b) outlets.

Table S1: Sensitivity and specificity of the outlets in 3- and 5-outlets devices

|  | **3-Outlets Device** | | **5-Outlets Device** | | | |
| --- | --- | --- | --- | --- | --- | --- |
|  | Soft outlet | Stiff outlet | Soft-1 outlet | Stiff-1 outlet | Soft-1+Soft-2 outlets | Stiff-1+Stiff-2 outlets |
| Specificity* (%) | 96.69 | 92.21 | 99.87 | 99.93 | 94.44 | 97.42 |
| Sensitivity^#^ (%) | 54.30 | 59.56 | 29.98 | 28.82 | 77.95 | 82.76 |

^*^$\%Specificity=\frac{True Negative}{True Negative+False Positive}$

^#^$\%Sensitivity=\frac{True Positive}{True Positive+False Negative}$


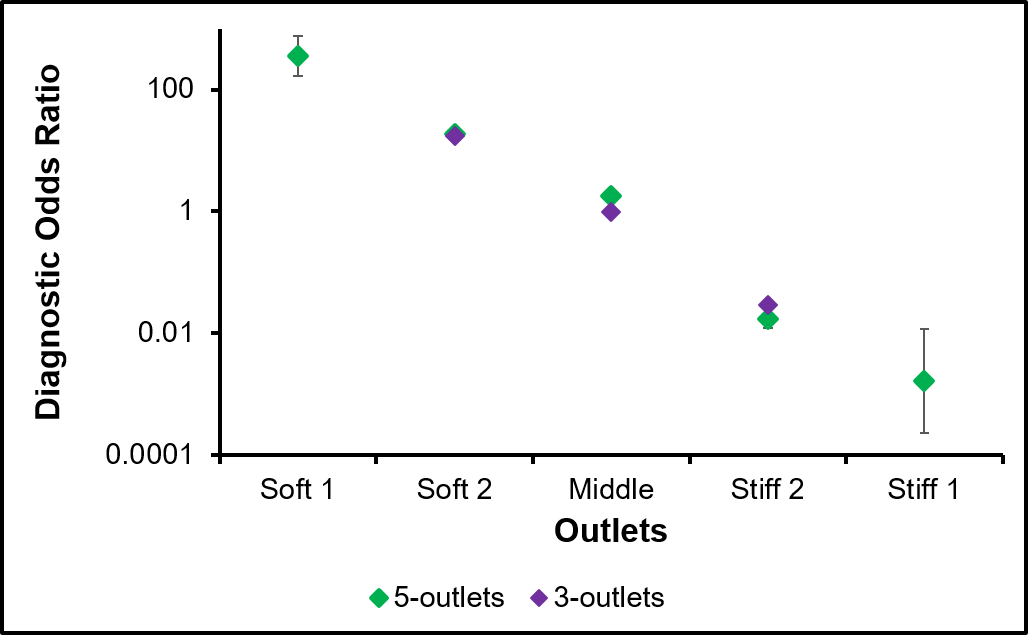


Fig. S2: Diagnostic odd ratio (DOR) for 3-outlets and 5-outlets devices. 5-outlets device has much higher DOR compared to 3-outlets device. The Error bars indicate the 95% confidence interval.


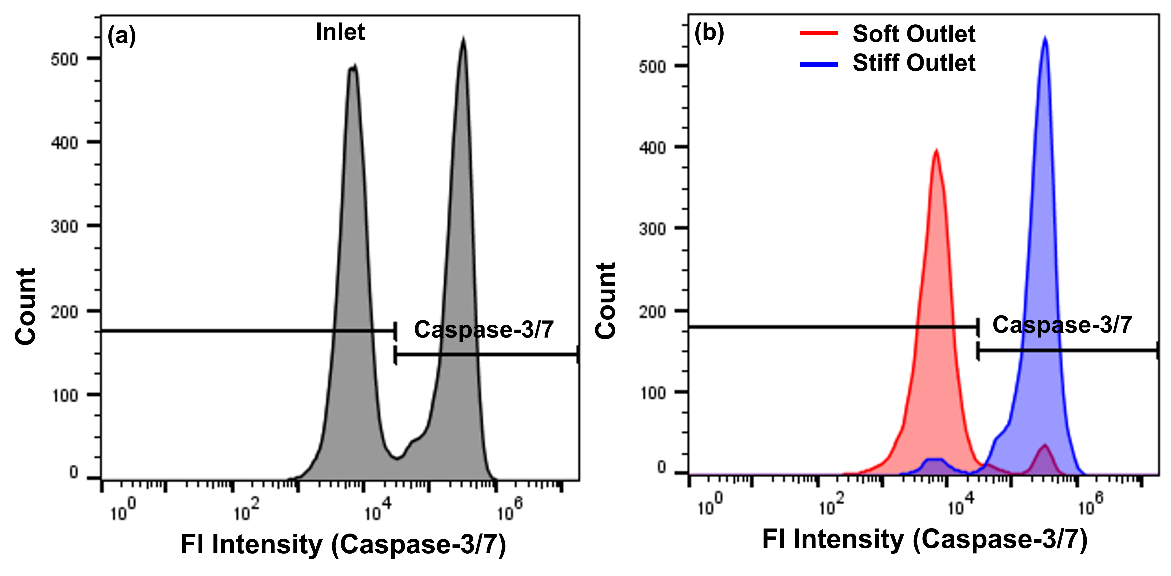


Fig. S3: Activity of Caspase-3/7 gene of the sorted cells (K562 cells treated with 2 µM daunorubicin). Flow cytometry data from (a) inlet; (b) outlets.


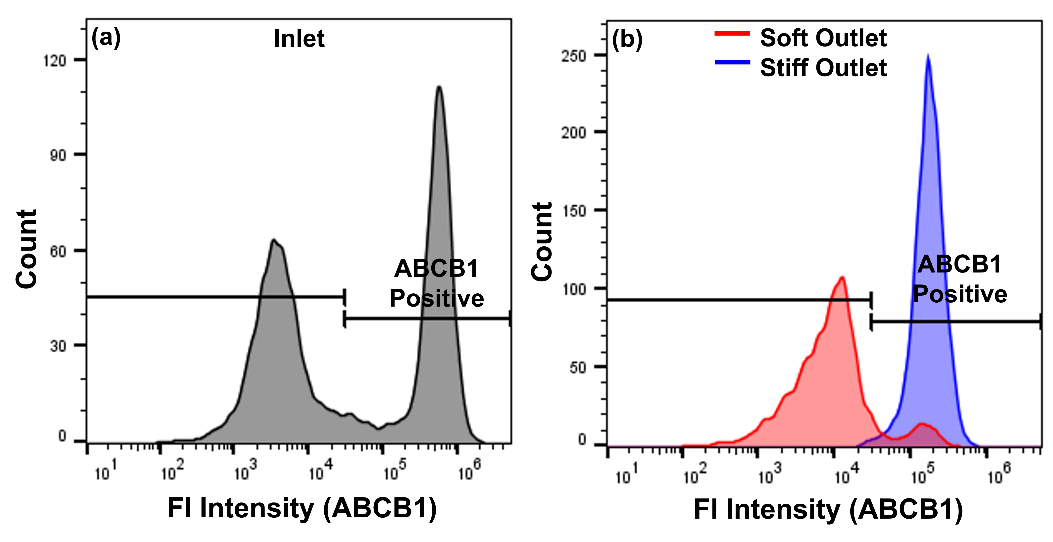


Fig. S4: Expression of ABCB1 gene of the sorted cells (K562 cells treated with 2 µM daunorubicin). Flow cytometry data from (a) inlet; (b) outlets.

Table S2: List of designed primers

| **GENE** | **Forward Primer** | **Reverse Primer** |
| --- | --- | --- |
| **ABCB1** | gctcctgactatgccaaagc | tcttcacctccaggctcagt |
| **FOXO3** | cccaaccagctcctttaaca | gaagtgagcaggtcctggag |
| **AKT-1** | acaaggacgggcacattaag | accgcacatcatctcgtaca |
| **Casp3** | tttttcagaggggatcgttg | cggcctccactggtatttta |
| **Casp7** | aagatcccagtggaagctga | tctcatggaagtgtgggtca |
| **KRT19** | tttgagacggaacaggctct | ccctcagcgtactgatttcc |
| **BCL-2** | gaggattgtggccttctttg | acagttccacaaaggcatcc |


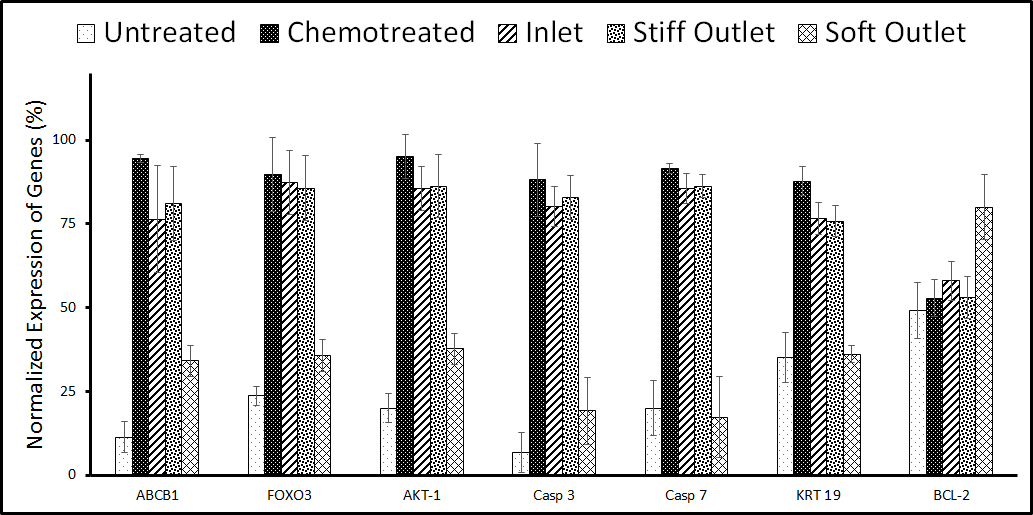


Fig. S5: Representative gene expression of untreated, chemotreated but unsorted, and chemotreated sorted cells from stiff and soft outlets obtained by polymerase chain reaction analysis.


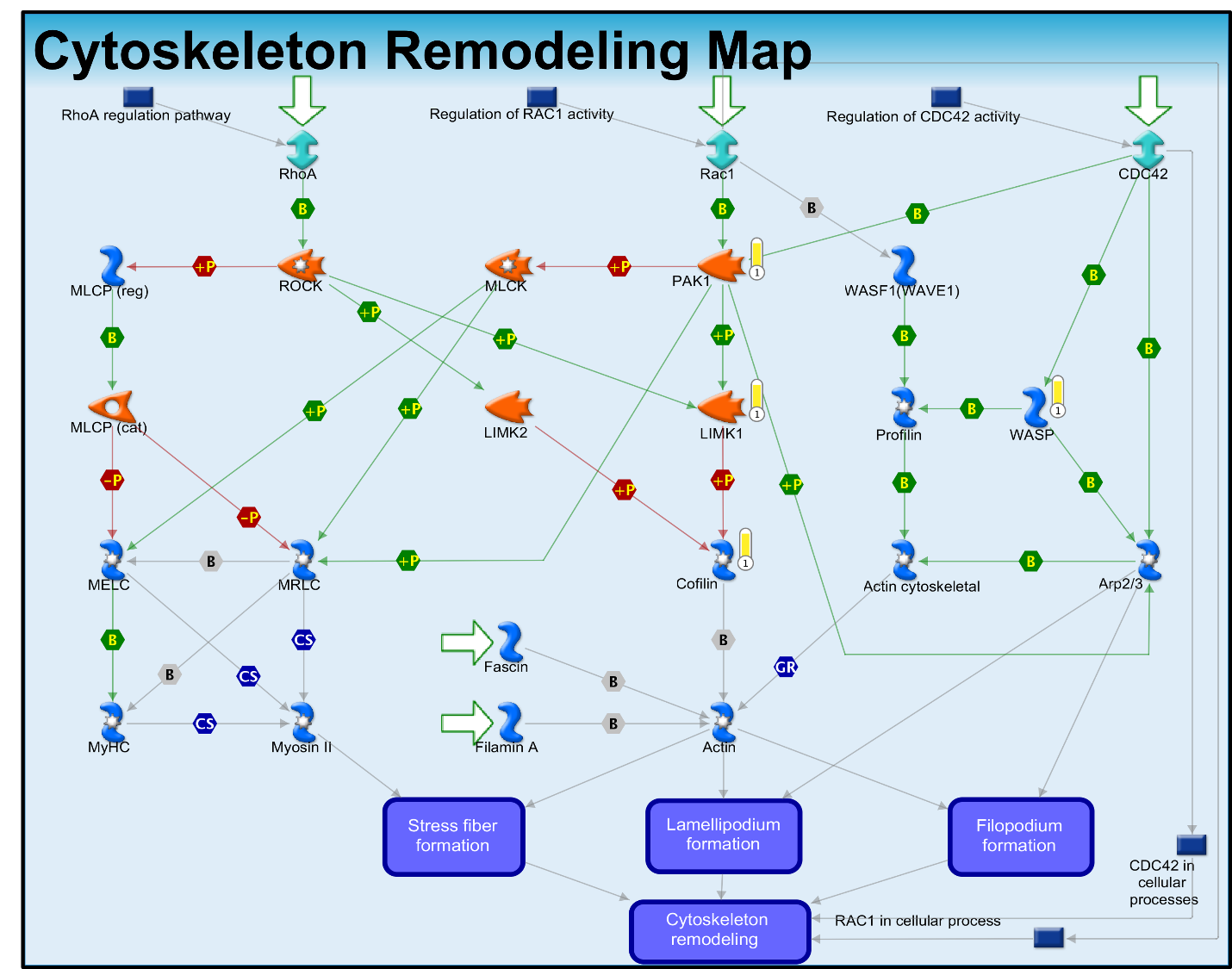


Fig. S6: Cytoskeleton remodeling. Regulation of actin cytoskeleton by Rho GTPases. Yellow thermometer: genes topologically relevant to set of up-regulated genes in soft (resistant) cells (for additional legend: <https://portal.genego.com/legends/MetaCoreQuickReferenceGuide.pdf>).


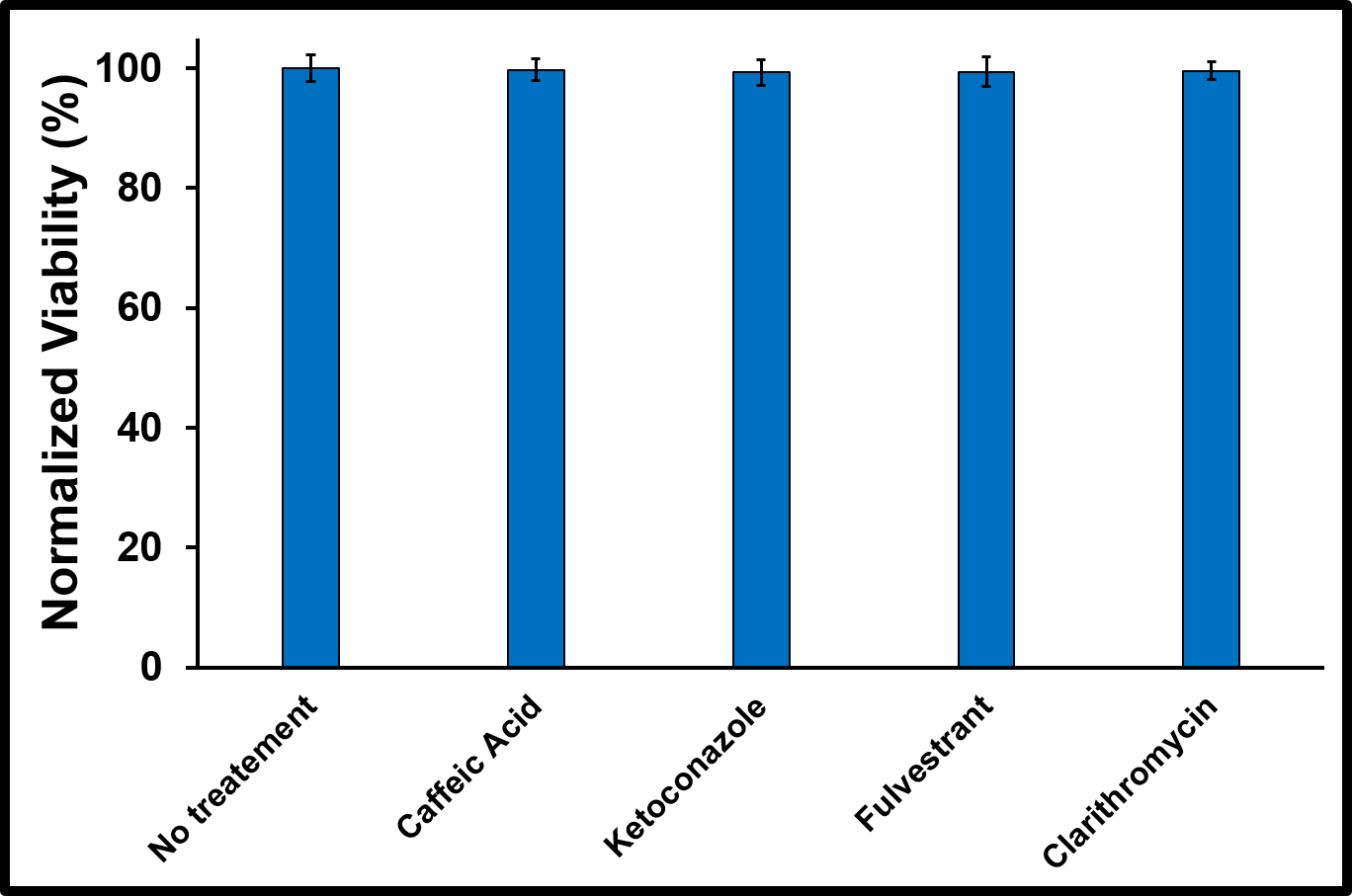


Fig. S7: Average viability (with standard deviation) of only inhibitors treated K562 cells (*N=4*).

**Gene Expression Pathways**

Up-regulation of CYP450 enzymes, however, can increase metabolic activation of other anticancer drugs and increase cell sensitivity by this mechanism. For instance, CYP3A4 and CYP2B6 are major monooxygenases catalyzing activation of anticancer drugs cyclophosphamide and ifosfamide through 4-hydroxylation reaction.(1) This finding suggests vulnerability of soft (resistant) cells to these specific anticancer drugs that are activated by over-expressed CYP450 monooxygenases.

In addition, higher ratio of bcl-2 to bax protein is associated with decreased rates of complete remission and reduced overall survival in acute myelogenous leukemia (AML).(2)

Up-regulation of ABCC1 induced by step-wise exposure of leukemia Jurkat cells to vincristine was previously shown to confer resistance to other antileukemic therapeutics daunorubicin and prednisone.(3)

EGFR is a prototypical growth factor receptor from HER family of transmembrane receptors binds more than 10 different ligands, which triggers signaling through Ras/mitogen-activated protein kinase (MAPK), phospholipase C (PLC)/protein kinase C (PKC), phosphatidylinositol 3-kinase (PI3K)/Akt, and the Jak–Stat signal amplification pathways that mediate various cellular responses, including proliferation, differentiation, migration, adhesion, and apoptosis.(4) Furthermore, up-regulation of EGFR has been found in 33% of human AML cases and several leukemia cell lines, including K562 cells, and its expression was reportedly associated with poor clinical outcomes.(5) In some cancers, inhibition of EGFR signaling led to promotion of apoptosis through activation of proapoptotic pathways, inhibition of antiapoptotic pathways, or through a combination of both mechanisms. For instance, inhibition of EGFR with chimeric (mouse/human) monoclonal antibody cetuximab suppressed cell proliferation, enhanced apoptosis and radiation sensitivity of squamous cell carcinoma cells from head and neck cancer patients, which was associated with increased Bax and decreased Bcl-2 expression.

Interpretation of the up-regulation of TOP2A and down-regulation of TP53 genes in context of observed drug resistance is not straightforward. In the case of TP53, a mutational inactivation of this gene in K562 cells through homozygous single base insertion between codons 135 and 136 results in production of truncated protein of 147 amino acids.(6) This mutation is expected to result in loss-of-function and the role of down-regulation of truncated p53 protein is not clear. Gene TOP2A display changes in expression, which are not consistent with increased resistance of cells to an anthracycline drug daunorubicin. Resistance to anthracyclines is reportedly associated with reduced expression of TOP2A,(7) whereas the soft (resistant) cells in our study displayed up-regulation of TOP2A. Perhaps, the positive influence of TOP2A on cell sensitivity to daunorubicin was compensated by inhibition of apoptosis through other expression changes associated with drug resistance and described previously. TOP2A up-regulation could be just a consequence of up-regulation of ESR1 (estrogen receptor 1; ERα) detected in softer cells, which would be consistent with previous reports, which identified positive influence of ESR1 on TOP2A expression.(8)

Other transcription factors, which may be responsible for the gene expression changes, include again PPARA and NF-kB, identified by interactome and differential expression analyses, but possibly also other transcription factors, such as hepatocyte nuclear factor 4 alpha (HNF4A), SP1, c-Jun, pregnane X receptor (PXR), and constitutive androstane receptor (CAR) (Supplemental file 2). As mentioned earlier, CYP4A4, which was found up-regulated in soft cells, is known to be regulated by PXR and CAR. However, CYP3A4 expression regulated by PXR or CAR is most prominent in the liver, where liver-specific transcription factor HNF4A is critically involved in PXR- and CAR-mediated induction of CYP3A4 by xenobiotics.(9) Consequently, we hypothesize that HNF4A, identified as overconnected to our set of differentially expressed genes, as well as transcription factor relevant to soft cells, is ectopically involved in facilitation of CYP3A4 induction by xenobiotic sensors PXR and/or CAR in soft cells. HNF4A-centric network (Fig. S8) was also identified among 30 prioritized transcription factor-centric networks for differentially expressed genes (Supplemental file 2). Our hypothesis on the involvement of HNF4A is supported by a study reported by *Ganesan et al.,* who detected expression of HNF4A in leukemia cells.(10)

Furthermore, activation of NF-κB is supported by the results of topological significance analysis of gene expression data. Submission of 24 up-regulated genes in soft vs stiff cells, in the form of Entrez Gene IDs, produced a list of 551 topologically significant genes, of which 549 were recognized by David Bioinformatic Resources 6.8 (https://david.ncifcrf.gov/) and assigned official gene symbols, which mapped to 814 MetaCore global interactome network objects. Enrichment analysis for these network objects in GeneGO canonical "Pathway Maps" functional ontology identified 460 pathway maps significantly enriched for genes topologically relevant to our differentially expressed dataset (Supplemental file 3). The list of significantly enriched maps includes "Development EGFR signaling pathway" (Supplemental file 4, Fig. S9), "Signal transduction NF-κB activation pathways" (Supplemental file 4, Fig. S10), and numerous cytoskeleton remodeling pathways, among which "Cytoskeleton remodeling_ESR1 action on cytoskeleton remodeling and cell migration" (Supplemental file 4, Fig. S11) implies the role of estrogen receptor signaling on actin cytoskeleton and possibly on the difference in cell stiffness observed in our study. Activation of estrogen receptor signaling by treatment with estradiol has been shown to induce cytoskeletal changes and decrease stiffness of cultured human fetal osteoblasts.(11) Similarly, estradiol was shown to decrease stiffness of endothelial cells through non-genomic ERβ pathways.(12)


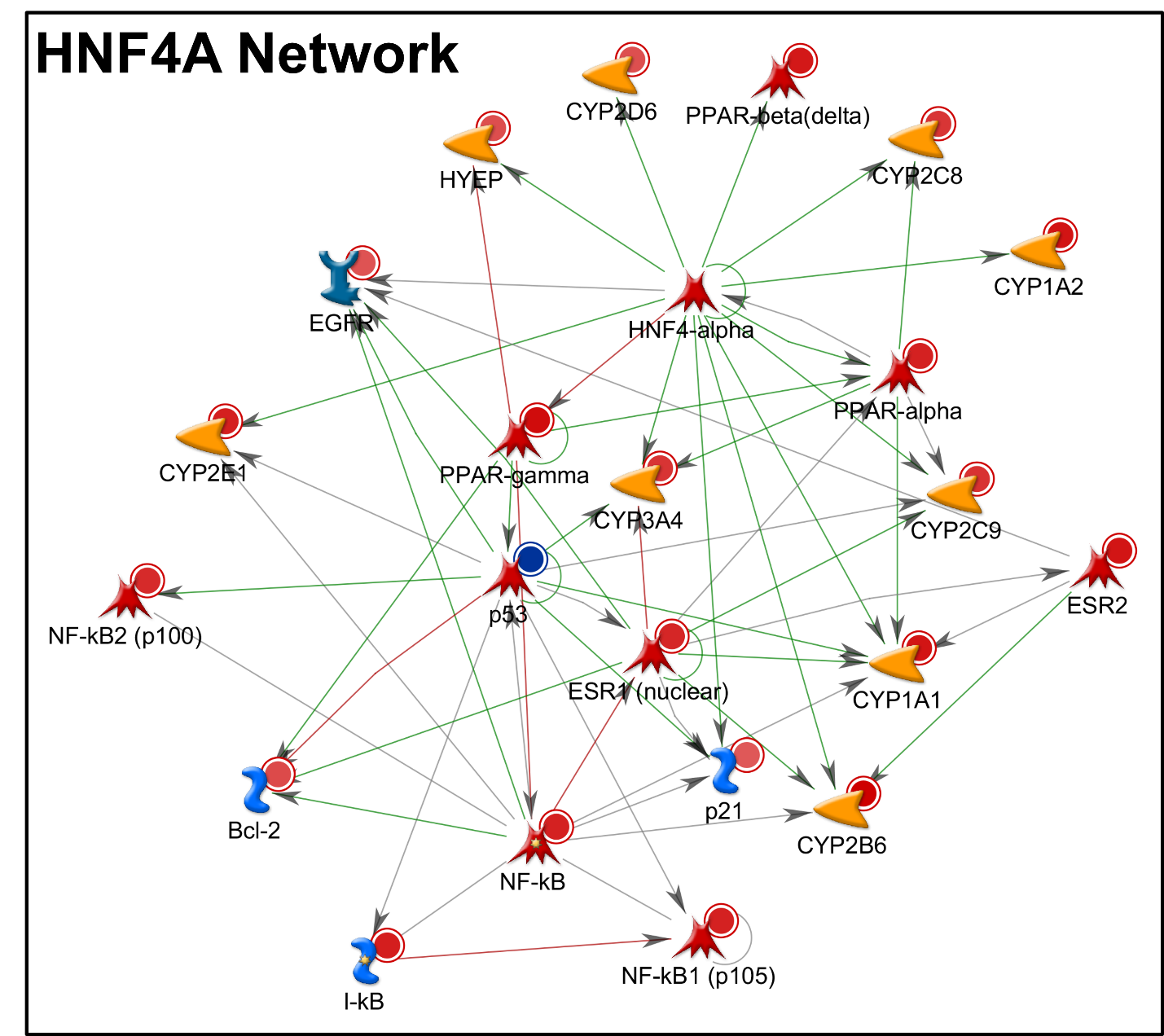


Fig. S8: Transcription factor-centric network around HNF4A build from genes differentially expressed between soft and stiff cells. Red circle: up-regulated gene; Blue circle: down-regulated gene; green edge: transcriptional activation; red edge: transcriptional repression (for additional legend: <https://portal.genego.com/legends/MetaCoreQuickReferenceGuide.pdf>).


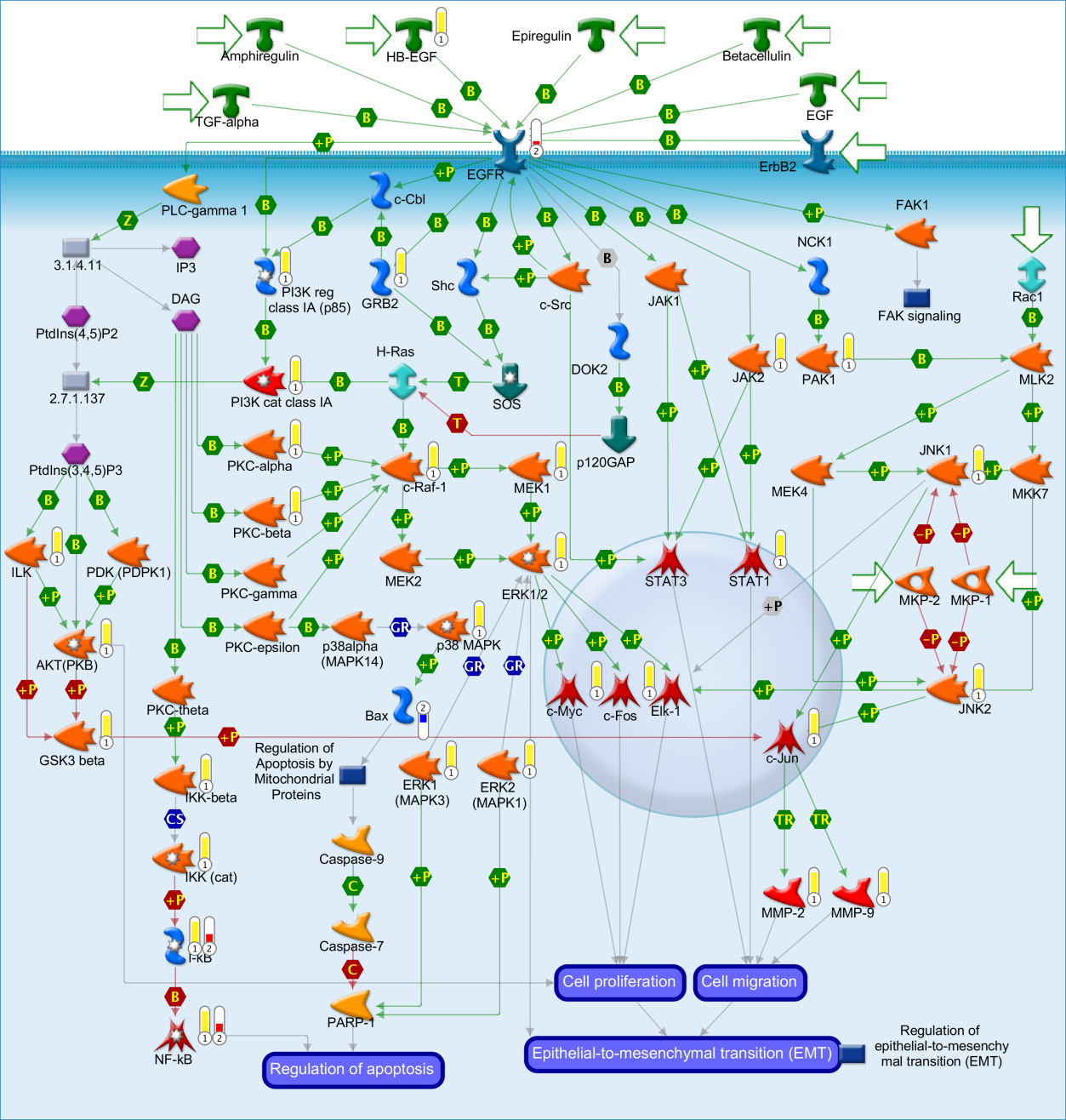


Fig. S9: **Development EGFR signaling pathway.** Red thermometer: genes up-regulated in soft (resistant) cells; blue thermometer: genes down-regulated in soft (resistant) cells; yellow thermometer: genes topologically relevant to set of up-regulated genes in soft (resistant) cells. For additional legend see: https://portal.genego.com/legends/MetaCoreQuickReferenceGuide.pdf


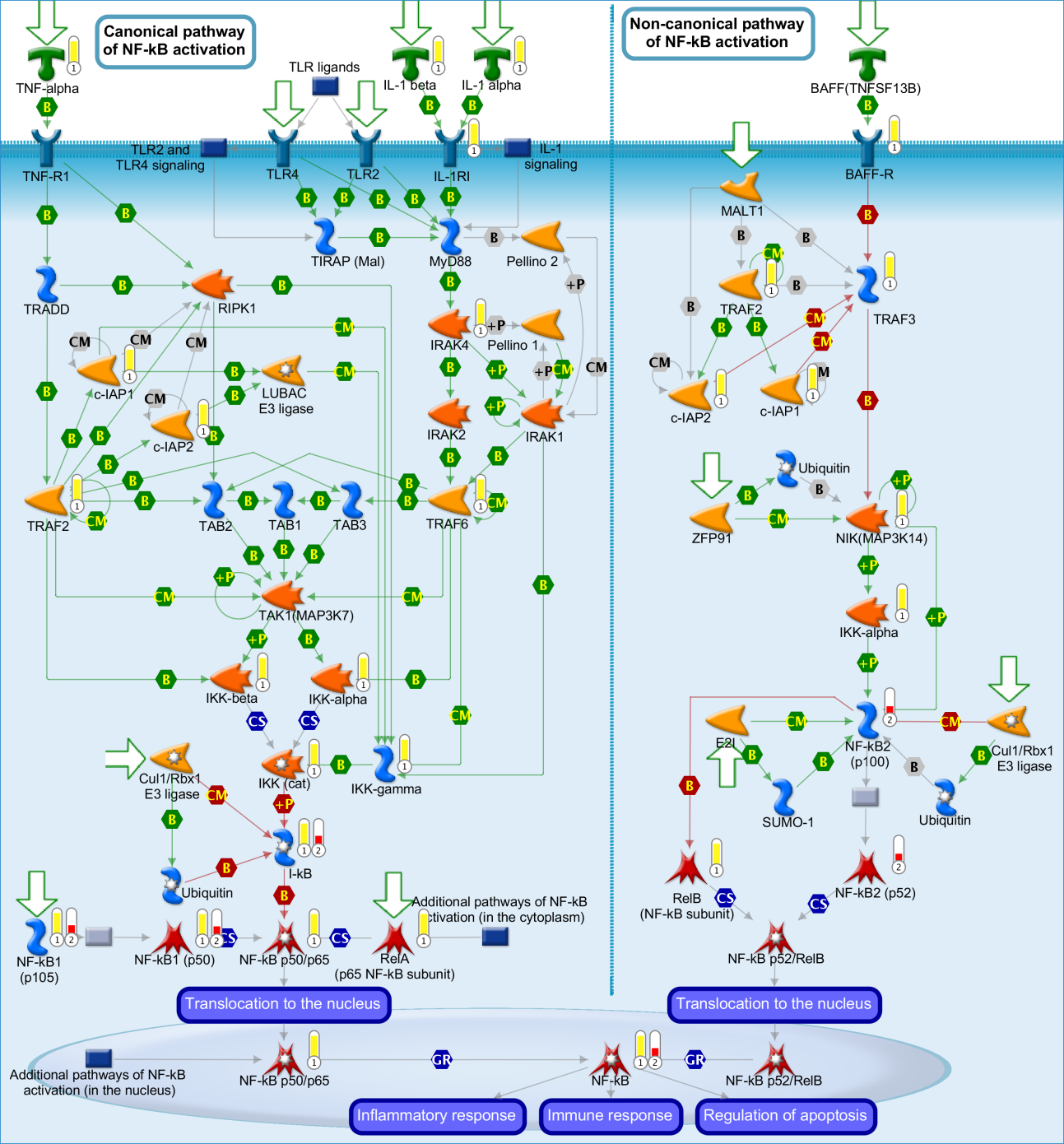


Fig. S10: **Signal transduction NF-κB activation pathways.** Red thermometer: genes up-regulated in soft (resistant) cells; blue thermometer: genes down-regulated in soft (resistant) cells; yellow thermometer: genes topologically relevant to set of up-regulated genes in soft (resistant) cells. For additional legend see: https://portal.genego.com/legends/MetaCoreQuickReferenceGuide.pdf


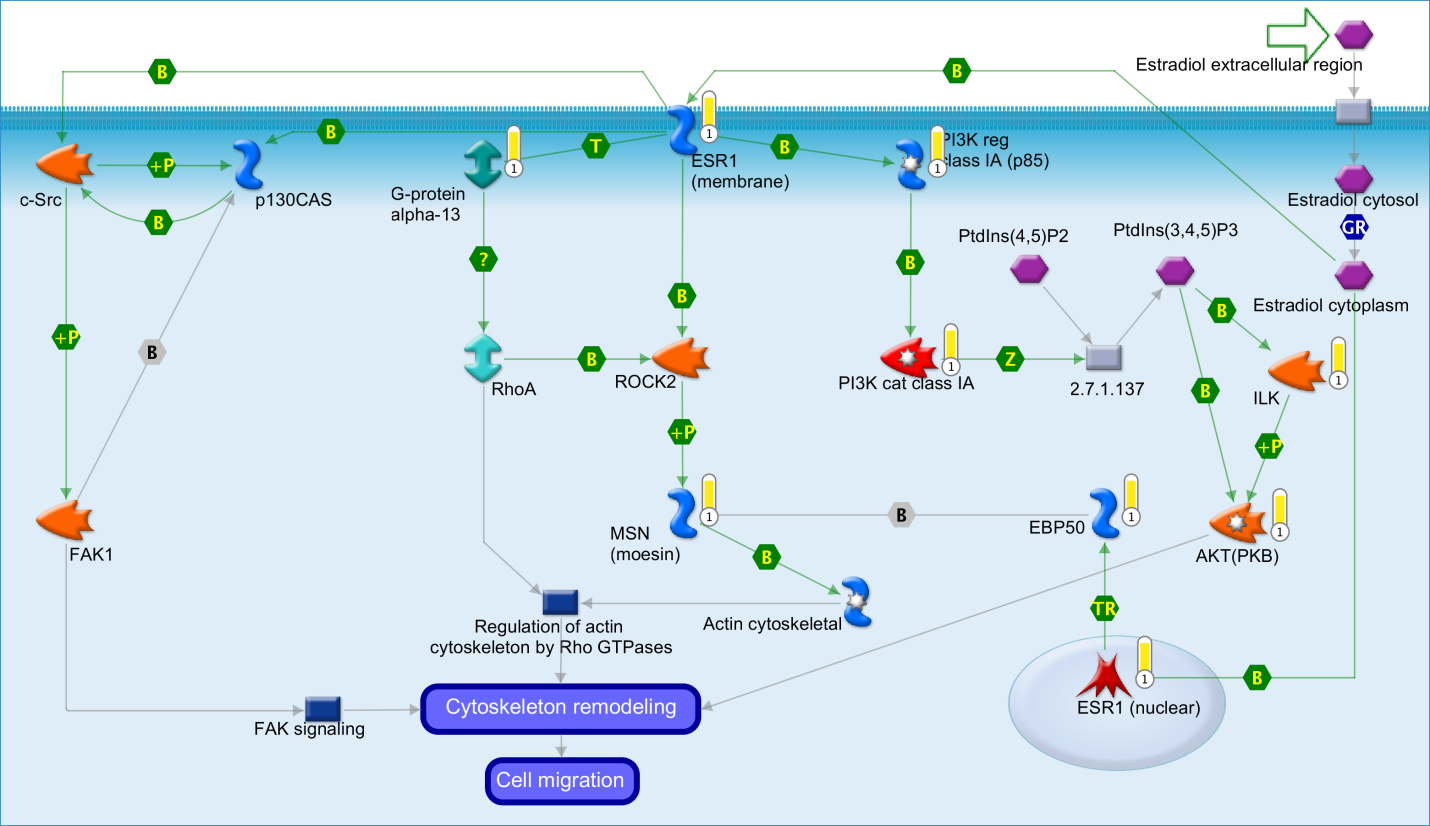


Fig. S11: **Cytoskeleton remodeling_ESR1 action on cytoskeleton remodeling and cell migration.** Yellow thermometer: genes topologically relevant to set of up-regulated genes in soft (resistant) cells. For additional legend see URL

<https://portal.genego.com/legends/MetaCoreQuickReferenceGuide.pdf>)

Table S3: Genes differentially expressed between soft (resistant) and stiff (sensitive) cells. *p*-value was calculated using paired t-test. *q*-values were determined using the package '*q*-value' (Bioconductor) (*N=2*).

| Genes | FC | p-val | q-val |
| --- | --- | --- | --- |
| CYP2B6 | 4.76 | 2.83E-02 | 6.47E-02 |
| PPARG | 4.32 | 3.24E-02 | 6.47E-02 |
| CYP1A1 | 4.05 | 1.80E-02 | 6.47E-02 |
| ESR2 | 4.01 | 2.44E-02 | 6.47E-02 |
| PPARD | 3.88 | 2.57E-02 | 6.47E-02 |
| NFKBIE | 3.66 | 4.14E-02 | 7.31E-02 |
| CYP2E1 | 3.62 | 1.11E-02 | 6.35E-02 |
| CYP2C19 | 3.61 | 1.52E-02 | 6.35E-02 |
| CYP1A2 | 3.6 | 1.39E-02 | 6.35E-02 |
| PPARA | 3.55 | 1.05E-02 | 6.35E-02 |
| NFKB1 | 3.53 | 3.12E-02 | 6.47E-02 |
| NFKBIB | 3.5 | 1.02E-02 | 6.35E-02 |
| ESR1 | 3.37 | 7.17E-03 | 6.35E-02 |
| NFKB2 | 3.2 | 1.33E-02 | 6.35E-02 |
| TOP2A | 3.01 | 2.66E-02 | 6.47E-02 |
| CYP2C9 | 2.91 | 3.21E-02 | 6.47E-02 |
| CYP3A4 | 2.84 | 4.04E-02 | 7.31E-02 |
| CYP2C8 | 2.79 | 3.79E-02 | 7.25E-02 |
| BCL2 | 2.03 | 1.23E-02 | 6.35E-02 |
| EPHX1 | 1.87 | 3.47E-03 | 6.35E-02 |
| CYP2D6 | 1.82 | 2.32E-02 | 6.47E-02 |
| ABCC1 | 1.81 | 7.85E-03 | 6.35E-02 |
| EGFR | 1.68 | 2.47E-02 | 6.47E-02 |
| CDKN1A | 1.49 | 4.33E-02 | 7.36E-02 |
| ABCB1 | -2.56 | 3.00E-02 | 6.47E-02 |
| BAX | -3.57 | 1.36E-02 | 6.35E-02 |
| TP53 | -5.26 | 1.84E-02 | 6.47E-02 |

**References:**

1. Kivisto KT, Kroemer HK, Eichelbaum M. The role of human cytochrome P450 enzymes in the metabolism of anticancer agents: implications for drug interactions. *Br J Clin Pharmacol.* 1995; **40:** 523-530.

2. Del Poeta G, Bruno A, Del Principe MI, Venditti A, Maurillo L, Buccisano F, et al. Deregulation of the mitochondrial apoptotic machinery and development of molecular targeted drugs in acute myeloid leukemia. *Current Cancer Drug Targets* 2008; **8:** 207-22.

3. Winter SS, Ricci J, Luo L, Lovato DM, Khawaja HM, Serna-Gallegos T, et al. ATP Binding Cassette C1 (ABCC1/MRP1)-mediated drug efflux contributes to disease progression in T-lineage acute lymphoblastic leukemia. *Health* 2013; **5:** 5A.

Allen GW, Harari PM. The advancement of epidermal growth factor recetor inhibitors in cancer therapy. In: Gewirtz DA, Holt SE, Grant S, editors. Apoptosis, senescence and cancer. *Cancer Drug Discovery and Development*. Totowa, New Jersey, USA: Humana Press; 2007; pp 335-357.

5. Sun JZ, Lu Y, Xu Y, Liu F, Li FQ, Wang QL, et al. Epidermal growth factor receptor expression in acute myelogenous leukaemia is associated with clinical prognosis. *Hematological Oncology* 2012; **30:** 89-97.

6. Law JC, Ritke MK, Yalowich JC, Leder GH, Ferrell RE. Mutational inactivation of the p53 gene in the human erythroid leukemic K562 cell line. *Leukemia Research* 1993; **17:** 1045-50.

7. Burgess DJ, Doles J, Zender L, Xue W, Ma B, McCombie WR, et al. Topoisomerase levels determine chemotherapy response in vitro and in vivo. *Proceedings of the National Academy of Sciences of the United States of America* 2008; **105:** 9053-8.

8. Tirino V, Desiderio V, Paino F, De Rosa A, Papaccio F, La Noce M, et al. Cancer stem cells in solid tumors: an overview and new approaches for their isolation and characterization. *The FASEB Journal* 2013; **27:** 13-24.

9. Tirona RG, Lee W, Leake BF, Lan LB, Cline CB, Lamba V, et al. The orphan nuclear receptor HNF4alpha determines PXR- and CAR-mediated xenobiotic induction of CYP3A4. *Nature Medicine* 2003; **9:** 220-4.

10. Ganesan S, Zhang W, Abraham A, Varatharajan S, Rajamani BM, Velayudhan SR, et al. Expression Profiling Of Nuclear Hormone Receptors In Myeloid Leukemia Reveals Potential Novel Drug Targets For Combination Therapy. *Blood* 2013; **122:** 3855.

11. Muthukumaran P, Lim CT, Lee T. Estradiol influences the mechanical properties of human fetal osteoblasts through cytoskeletal changes. *Biochemical and Biophysical Research Communications* 2012; **423:** 503-8.

12. Hillebrand U, Lang D, Telgmann RG, Hagedorn C, Reuter S, Kliche K, et al. Nebivolol decreases endothelial cell stiffness via the estrogen receptor beta: a nano-imaging study. *Journal of Hypertension* 2009; **27:** 517-26.

1. RM's current affiliation includes Office of Research and Development, US EPA. The views expressed in this paper are those of the author(s) and do not represent official EPA policy. [↑](#footnote-ref-1)
